# Synergistic Combination of *Cryptolepis sanguinolenta (Lindl.)* Schlechter (Apocynaceae) and *Azadirachta Indica* (A. Juss) (Meliaceae) Against 3D7 and DD2 Strains of *Plasmodium Falciparum*

**DOI:** 10.1155/adpp/1273876

**Published:** 2026-08-31

**Authors:** Samuel Korsah, Nathaniel Nene Djangmah Nortey, Christian Akakpo, Felix Kwame Zoiku, Jessica Korsah, Miriam Tagoe, Joseph Kwao Amanor, Babu Jacob Yamba, Josephine Akosua Ofori-Asamoah

**Affiliations:** ^1^ Department of Pharmaceutical Sciences, School of Pharmacy, Central University, Accra, Ghana, central.edu.gh; ^2^ Department of Biology, University of New Brunswick, Fredericton, Canada, unb.ca; ^3^ Department of Natural Product Sourcing and Herbarium, Institute of Traditional and Alternative Medicine, University of Health and Allied Sciences, Ho, Ghana, uhas.edu.gh; ^4^ Noguchi Memorial Institute for Medical Research, University of Ghana, Accra, Ghana, ug.edu.gh

**Keywords:** antimalarial, *Azadirachta indica*, combination index, combination therapy, *Cryptolepis sanguinolenta*, dose reduction index, isobologram, resistant strains, synergism

## Abstract

**Introduction:**

*Azadirachta indica* and *Cryptolepis sanguinolenta* are herbs commonly used together in polyherbal products in Ghana for the treatment of malaria.

**Aim:**

This research investigated the combined effects of *Azadirachta indica* and *Cryptolepis sanguinolenta* extracts against chloroquine‐sensitive (3D7) and resistant (DD2) strains of *Plasmodium falciparum*.

**Methods:**

In vitro antimalarial activity was assayed on the aqueous extracts of both plants using a fluorescence‐based SYBR Green assay. The combined effects of the two extracts were determined using computer‐based software, CompuSyn.

**Results:**

Aqueous extracts of *Azadirachta indica* and *Cryptolepis sanguinolenta* exerted high antimalarial activities (IC_50_ ≤ 10 μg/mL) against both 3D7 and DD2 strains of the *Plasmodium falciparum*. *Azadirachta indica* and *Cryptolepis sanguinolenta* displayed strong synergistic activity with combination index (CI) values ranging from 0.15 to 0.52 for 53%–99% inhibition of 3D7 *Plasmodium falciparum*. Also, CI values for the plants against DD2 were from 0.23 to 0.61 with corresponding 30%–97% parasitemia inhibitions. *Azadirachta indica* and *Cryptolepis sanguinolenta* exerted a dose reduction index (DRI) value greater than 1 (DRI > 1) in both strains.

**Conclusion:**

*Cryptolepis sanguinolenta* and *Azadirachta indica* exerted synergistic interactions against 3D7 and DD2 strains of *Plasmodium falciparum*. The extracts are desirable when used in combination rather than alone.

## 1. Introduction

Malaria is a global health issue which affects millions of people around the world annually [1]. It is transmitted through the bite of the female Anopheles mosquito infected with a parasite of the genus *Plasmodium*. Four species of protozoa of the genus *Plasmodium* can cause human malaria: *Plasmodium falciparum*, *Plasmodium vivax*, *Plasmodium malariae* and *Plasmodium ovale* [2]. Among these, *Plasmodium falciparum* is responsible for the most complicated and lethal cases, and together with *Plasmodium vivax*, they represent over 95% of all malaria cases [3]. The reproduction and development of Anopheles mosquitoes are enhanced in the tropical climate; hence, malaria thrives, and it is widespread in the region. According to the World Malaria Report [4], 229 new malaria cases and 409,000 malaria deaths worldwide were recorded, with a mortality rate of 95% in 31 European countries [5]. Malaria is present in more than 90 countries globally; 36% of the world’s population lives in areas where malaria transmission is a possibility, 7% lives in areas where malaria has never been effectively controlled, and 29% lives in areas where malaria was previously transmitted at low levels or not at all but where significant transmission has been re‐established, according to the data provided by the World Health Organisation [4].

The use of herbs in various treatments is increasing due to many reasons. People in traditional and rural communities resort to herbal preparations to treat malaria due to the availability of these materials [6]. Also, natural remedies are considered safe and more effective compared to allopathic medicine. Recent studies suggest that parasite resistance to current treatment is inevitable [5]. One of the biggest obstacles to the current control of malaria is the emergence and spread of drug‐resistant forms of the parasite [7]. Resistance to the quinine antimalarial drug was first noted in the 1910s. Parasites’ resistance to chloroquine was discovered in the 1950s. Also, *Plasmodium* parasites’ resistance to pyrimethamine was observed in the same year when the drug was introduced to the market. Reports of parasite strains resistant to the currently used artemisinin and its derivatives present a huge challenge as well [8].

One of the strategies widely used to overcome the evolution of resistance is combination therapy [9]. The use of synergistic drugs inhibits both sensitive and resistant strains and arrests the selective power of resistant evolution. Synergistic drugs also lead to remarkable therapeutic effects with low doses and less toxicity, that is, achieving more with less. Synergy is demonstrated by a modified Loewe additivity model as isobologram‐combination index (Fa‐CI) by [10, 11]. This comprises the median‐effect equation from the unified theory mass‐action law principle to calculate the CI using both the median‐effect equation and dose‐effect relationships of individual drugs and their combinations. This is represented as: CI $=D_{1}/{\left(D_{1}-fa\right)}_{1}+D_{2}/{\left(D_{1}-fa\right)}_{2}+\alpha \left(D_{1}\right)\left(D_{2}\right)/{\left(D_{1}-fa\right)}_{1}$, where *D*_1_ and *D*_2_ are doses of the drugs in combination, *D*_1_‐*fa* are doses of the individual drugs needed to achieve the same effect (fa) and *α* is 0 for mutually exclusive drugs and 1 for mutually nonexclusive drugs.

*Azadirachta indica* and *Cryptolepis sanguinolenta* are herbs that have been frequently used in parts of Africa and Southeast Asia in the treatment of malaria for over a hundred years. Numerous publications have documented the antiplasmodial activities of both plants. In our previous works, we reported on plants that are mostly used in polyherbal formulations in parts of Ghana and ranked them based on the frequency of usage in many herbal products [12]. We subsequently selected the top six most frequently used herbs and screened for their individual antiplasmodial activities against 3D7 and DD2 *Plasmodium falciparum* strains [13]. *Cryptolepis sanguinolenta* and *Azadirachta indica* are the most effective antimalarial herbs among the frequently used six plants screened. This current research further investigated the combined effects of *Azadirachta indica* and *Cryptolepis sanguinolenta* against chloroquine‐sensitive (3D7) and resistant (DD2) strains of *Plasmodium falciparum* using CompuSyn software. We used dose‐response, median effect, CI, dose reduction index (DRI) and isobolograms to assess the combination effects of these plants.

## 2. Methodology

### 2.1. Materials

#### 2.1.1. Sample Collection

The samples *A. indica* were collected from Ayikuma (5.9,491,684,−0.0164631) and *C. sanguinolenta* from Mampong (5.9,186,450,−0.1,338,355) in April 2024. The plant samples were identified and authenticated by Miss Mariam (botanist, Central University). After which, voucher numbers CUC/R/AM/002 and CUC/L/A/009 were assigned to *Cryptolepis sanguinolenta* and *Azadirachta indica,* respectively.

#### 2.1.2. Plasmodium Species

Chloroquine‐resistant (CQR) *Plasmodium falciparum* DD2 strain and chloroquine‐sensitive (CQS) *P. falciparum* 3D7 strains were obtained from the Department of Epidemiology at Noguchi Memorial Institute for Medical Research, University of Ghana and the Department of Biochemistry of the University of Ghana.

#### 2.1.3. Antimalarial Control Drugs and Reagents

Drugs and reagents used are: Artesunate (ATS) powder, dimethyl sulfoxide (DMSO) (sigma), HEPES, L‐glutamine, RPMI1640 powder (GIBCO) 10.43 g per pack, albumax, sodium bicarbonate, hypoxanthine, RPMI 1640 medium, glucose, albumax, hypoxanthine, SYBR Green 1 (10,000 in DMSO), immersion oil, Giemsa stain, ethanol, methanol, EDTA, Saponin, Triton X‐100, Gas mixture, Na_2_HPO4, NaCl, KH_2_PO4, Sorbitol, Glycerol, Gas mix (92.5% N_2_, 5.5% CO_2_, 2%O_2_).

#### 2.1.4. Equipment

Freeze dryer (Labconco Freezone 4.6 L), Class 11 biosafety cabinet, centrifuge, cell counter, vortex, aspirators, water bath, modular incubator chamber, CO_2_ incubator set at 37°C, electric vacuum pump, fluorescence plate reader (MTPR), light microscope, refrigerator, freezer were used to carry out the studies.

### 2.2. Plant Preparation and Extraction

Plant materials were washed thoroughly under running water, air‐dried for seven days and milled into coarse powder. For decoction, 900 g of each powdered sample was extracted with 5 L of distilled water. *A. indica* leaves were boiled for 15 min, whereas *C. sanguinolenta* roots were decocted for 30 min at 100°C. The extracts were cooled, filtered, and concentrated by rotary evaporation, followed by freeze‐drying at the Noguchi Memorial Research Institute. This process yielded 32.5 g of *A. indica* leaf extract and 43.4 g of *C. sanguinolenta* root extract. Dried extracts were stored at 4°C until use. This procedure was adopted from previous studies described by [13].

### 2.3. Phytochemical Profiling of Plant Materials

#### 2.3.1. Phytochemical Analysis

The dried powdered samples were used for phytochemical analysis to detect the secondary plant metabolites present using standard methods. Phytochemicals tested for include tannins, alkaloids, glycosides, saponins, flavonoids, triterpenoids and coumarins [14].

### 2.4. In Vitro Parasite Culturing and Preparation

**FIGURE 1:**
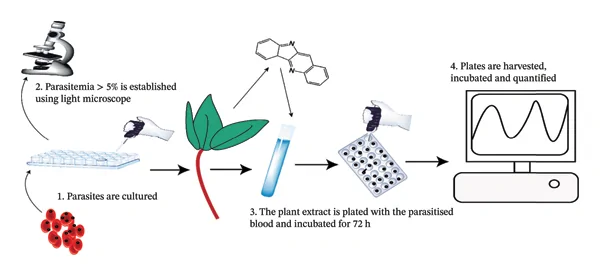
Summarised illustration of the processes in the in vitro antimalarial assay of a plant extract. Generated using bio instruments in chemdraw (Version 20.0) and adobe illustrator.

This study investigated the efficacy of various extracts against the asexual stages of *Plasmodium falciparum*, the malaria parasite. Continuous in vitro cultures of asexual‐stage *Plasmodium falciparum* were established and maintained using a modified protocol based on the method originally described by [15] (Figure 1). Cultures were sustained under a precisely controlled gaseous atmosphere comprising 93% nitrogen (N_2_), 4% carbon dioxide (CO_2_), and 3% oxygen (O_2_) at a constant temperature of 37°C, conditions designed to closely replicate the intraerythrocytic environment of the human host. The complete culture medium consisted of RPMI 1640 (10.44 g/L), HEPES (5.94 g/L), AlbuMAX II (5 g/L), hypoxanthine (50 mg/L) and sodium bicarbonate (2.1 g/L), supplemented with O‐positive human erythrocytes. Culture medium was replaced daily to maintain optimal nutrient availability and pH stability.

Parasite growth was monitored by preparing thin blood smears from cultures at regular intervals, staining with 10% Giemsa solution, and examining under oil immersion at × 100 magnification using a light microscope. Parasitaemia, defined as the percentage of *Plasmodium falciparum*‐infected erythrocytes relative to total red blood cells, was recorded until cultures attained a parasitaemia exceeding 5%, with a predominance of ring‐stage parasites, which is the earliest intraerythrocytic developmental form.

Prior to drug treatment, parasite cultures were synchronised by treatment with 5% D‐sorbitol to selectively lyse trophozoite and schizont‐stage‐infected erythrocytes, thereby enriching the culture for ring‐stage parasites and ensuring a developmentally homogeneous population for subsequent assays. A standardised inoculum was then prepared by adjusting cultures to 1% parasitaemia at 2% haematocrit in complete culture medium to a total volume of 14 mL. This standardised parasite suspension was used uniformly across all experimental conditions for the evaluation of the antimalarial activity of the test extracts.

### 2.5. Plating Parasites With Drug Compounds

The antimalarial activity of *Azadirachta indica* and *Cryptolepis sanguinolenta* extracts (CSEs) against *Plasmodium falciparum* strains 3D7 and DD2 had previously been determined using a fluorescence‐based SYBR Green assay following the protocol described by [13], which yielded half‐maximal inhibitory concentration (IC_50_) values of 24.48 μg/mL and 20.48 μg/mL, respectively. Working solutions of both extracts at their respective IC_50_ concentrations were prepared in complete culture medium supplemented with 0.5% DMSO as previously described [16] and subsequently used for downstream experimental procedures.

To assess the dose‐dependent effect of each extract on intraerythrocytic parasite development, average parasitaemia expressed as the proportion of *Plasmodium falciparum*‐infected erythrocytes relative to total red blood cells was determined by light microscopy following 72 h of continuous drug exposure. Six serial concentrations were derived from the previously established IC_50_ values of each extract by applying defined dilution factors (0 ×, 0.0625 ×, 0.125 ×, 0.25 ×, 0.5 × and 1 × IC_50_), yielding final test concentrations of 0, 1.53, 3.06, 6.12, 12.24 and 24.48 μg/mL for *A. indica*, and 0, 1.28, 2.56, 5.12, 10.24 and 20.48 μg/mL for *C. sanguinolenta*. Each concentration was evaluated against both *P. falciparum* strains (3D7 and DD2) in independent triplicates within 48‐well culture plates to ensure experimental accuracy and data reproducibility.

### 2.6. Antiplasmodial Effect of Combined Extracts

The second phase of the experiment assessed the combinatorial antimalarial effect of *Azadirachta indica* and *Cryptolepis sanguinolenta* aqueous extracts on *Plasmodium falciparum* parasitaemia. The six concentrations previously derived for each extract through twofold serial dilutions based on their respective IC_50_ values (0.25 ×, 0.5 ×, 1 ×, 2 × and 4 × IC_50_) were combined in a fixed 1:1 ratio (v/v), yielding the following paired combination concentrations of *A. indica* and *C. sanguinolenta*, respectively: 0:0, 1.305:1.53, 2.61:3.06, 5.22:6.12, 10.44:12.24 and 20.88:24.48 μg/mL.

Each combination concentration was dispensed in triplicate into 48‐well culture plates containing synchronised ring‐stage *P. falciparum* parasites at 1% initial parasitaemia in a total volume of 100 μL, maintained at 2% haematocrit in complete culture medium. Plates were transferred into a modular incubation chamber and incubated at 37°C for 72 h under standard gaseous conditions. Following the incubation period, cultures were harvested, and thin blood smears were prepared from each triplicate well for every concentration tested. Smears were fixed, stained with 10% Giemsa solution, and examined under oil immersion at × 100 magnification using a light microscope. Average percentage parasitaemia was determined by counting the proportion of infected erythrocytes per 1000 red blood cells per slide.

The average percentage of parasitaemia was subsequently calculated for both the single‐agent and combination treatment groups across the concentration series, for the chloroquine‐sensitive 3D7 and CQR DD2 strains of *P. falciparum*. Wells treated with the zero‐concentration vehicle control (0 μg/mL) served as the negative control for all experimental conditions, providing a baseline reference for parasitaemia under drug‐free conditions.

### 2.7. Combination Effect Analysis

**FIGURE 2:**
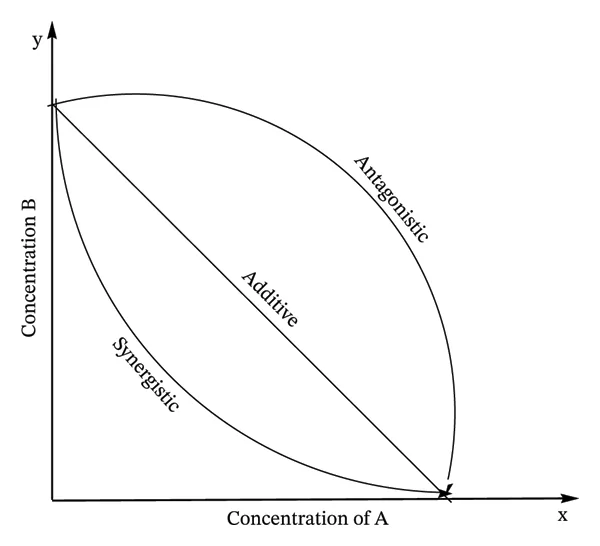
Graphical representation of isobologram.

The combined effects of the two extracts were determined using computerised software: CompuSyn (Version 1.0). The combination ratio for *Cryptolepis sanguinolepta* + *Azadirachta indica* (CS & AZ) was 1:1, with effects for both monotherapy and combinations expressed from 0 to 1, where 0: no effect and 1: highest effect (100%). Detailed procedures of automated dose‐effect analysis for simulation of synergism or antagonism are given in the User’s Guide for CompuSyn [17]. All data entry and report generation were done based on the steps described in [18]. Fa‐CI plot, DRI plot and isobologram plot were generated and used to characterise the combination effects of CS&AZ (Figure 2). The CI is a measure of the combination effect. CI = 1, < 1 and > 1 indicate additive effect, synergism and antagonism, respectively. DRI was calculated from the DRI equation and algorithm using CompuSyn software. DRI = 1, > 1 and < 1 indicate no dose reduction, favourable dose reduction and not favourable dose reduction, respectively, for each drug in the combination.

Interpreting the plot:

a. Additive: The plotted dots fall directly on the line of additivity, meaning the drugs simply combine to produce an expected 1 + 1 = 2 effect, showing no interaction.
b. Synergism: The plotted combination dots fall below the line, indicating you need smaller doses of both drugs to achieve the desired effect.
c. Antagonism: The plotted combination dots fall above the line, indicating larger doses are needed because the drugs interfere with one another.

### 2.8. Statistical Analysis

The average percentage parasitaemia of the strains in the culture was recorded before and after administering doses of *Cryptolepis sanguinolenta* and *Azadirachta indica* individually and as a combination drug. All experiments were done in triplicate, and results were presented as the standard error of the mean (SEM).

## 3. Results

### 3.1. Phytochemicals Detected in Plant Samples

The *A. indica* leaves and the roots of *C. sanguinolenta* had a percentage yield of 3.6% and 4.8%, respectively.

Phytochemical studies conducted on the powdered samples of both plants show that saponins and phytosterols were not detected in the powdered roots of *C. sanguinolenta*. All phytochemicals tested were detected in the powdered leaves of *A. indica* except coumarins (Table 1).

**TABLE 1:** Phytochemistry of *C. sanguinolenta* roots and *A. indica* leaves.

Test	Plant sample	
	*C. sanguinolenta*	*A. indica*
Flavonoids	+	+
Tannins	+	+
Glycosides	+	+
Saponins	—	+
Coumarins	+	—
Alkaloids	+	+
Sterols	—	+

*Note:* Key, Detected (+); Not Detected (−).

### 3.2. Antiplasmodial Activity of CSE and *Azadirachta Indica* Extract (AZE)

**FIGURE 3:**
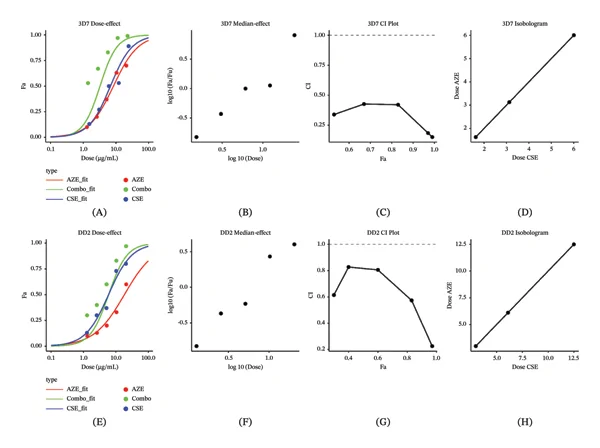
Comparative Chou–Talalay synergy analysis of CSE, AZE, and their fixed‐ratio combination in plasmodium falciparum 3D7 and DD2 strains. (A–D) 3D7 strain: (A) Dose‐effect curves for CSE, AZE, and the fixed‐ratio combination fitted using the median‐effect equation. (B) Median‐effect plot showing linearisation of the dose–response relationship and estimation of Dm and slope (m) parameters. (C) Combination index (CI) plot across fractional effect levels (Fa), demonstrating the interaction profile of the CSE–AZE combination, where CI < 1 indicates synergism. (D) Isobologram comparing experimental combination doses with the theoretical line of additivity at corresponding effect levels. (E–H) DD2 strain: (E) Dose–effect curves for CSE, AZE, and the combination. (F) Median‐effect plot showing the dose–response linearisation and derived Dm and m values. (G) CI plot across Fa levels illustrating the degree of synergism or additivity. (H) Isobologram demonstrating the interaction pattern of the combination relative to the additive line. Together, these analyses characterise the potency and interaction dynamics of CSE and AZE across chloroquine‐sensitive (3D7) and multidrug‐resistant (DD2) parasite backgrounds. All graphs were generated by CompuSyn software (Version 1) and re‐plotted using R (Version 2026.05.1); RScript is provided in the supporting document.

Table 2 shows the IC_50_ of CSE, AZE and ATS against 3D7 and DD2 lab strains. CSE and AZE displayed significant antiplasmodial activities with IC_50_ of 6.12 ± 0.5 μg/mL and 5.22 ± 0.6 μg/mL, respectively, against the 3D7 lab strain; 5.12 ± 0.7 μg/mL and 5.22 ± 0.6 μg/mL, respectively, against the DD2 lab strain. The asexual stages of the *Plasmodium falciparum* in a Giemsa‐stained slide are shown in Figure 2. The graphic representations obtained from the CompuSyn report for *Azadirachta indica* (AZE), *Cryptolepis sanguinolenta* (CSE), individually and combined (CSE–AZE) against the 3D7 and DD2 strains of *Plasmodium falciparum* are illustrated in Figure 3.

**TABLE 2:** IC_50_ values of the extracts and standard drug against 3D7 and DD2 strains of *Plasmodium falciparum*.

Extracts	IC_50_ ± SEM (μg/mL)	
	3D7	DD2
CSE	6.12 ± 0.5	5.12 ± 0.6
AZE	5.22 ± 0.6	5.12 ± 0.7
ATS	0.003 ± 9e–005	0.005 ± 2e–005

*Note:* IC_50_ values are expressed as mean ± SEM based on independent biological replicates.

Key for graphs:

AZE—*Azadirachta indica* (μg/mL).

CSE—*Cryptolepis sanguinolenta* (μg/mL).

CSE‐AZE—*Cryptolepis sanguinolenta* [mcg/mL] and *Azadirachta indica* (μg/mL) [1:1].

### 3.3. Combination Effect of CSE and AZE Against 3D7 and DD2 Lab Strains

**FIGURE 4:**
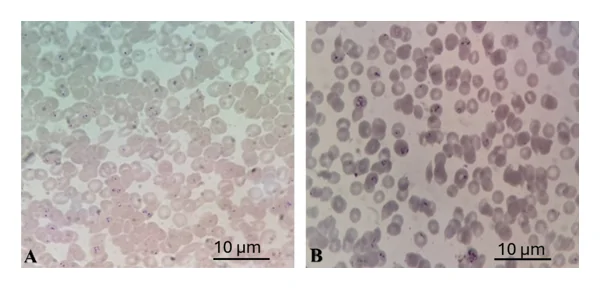
Plate 1: Asexual blood‐stage parasites of plasmodium falciparum 3D7 (A) and DD2 (B) visualised on Giemsa‐stained thin blood smears. Each panel shows infected and uninfected erythrocytes with characteristic intraerythrocytic parasite forms. The stained parasites appear as distinct purple chromatin dots and cytoplasmic rings within red blood cells, while uninfected erythrocytes display the expected pale pink cytoplasm and clear central pallor. Differences in staining intensity and parasite density between panels A and B reflect strain‐specific morphology and smear preparation.

With a CI less than 1 (0.34, 0.43, 0.42, 0.42, 0.18 and 0.15), CSE and AZE produced a highly synergistic effect against the *P. falciparum* 3D7 strain, as seen in Table 3 and Figure 4. The Dose Reduction Index [(5.31; 6.63), (4.11; 5.46), (4.01; 5.84) and (8.46; 15.03)] shows a favourable dose reduction; hence, the drugs could be used at a lower dose to achieve the same effect (Table 3).

**TABLE 3:** Synergy summary of CSE–AZE combination against *Plasmodium falciparum* 3D7 lab strain.

Combination dose (CSE + AZE, μg/mL)	Fa	CI	DRI (CSE; AZE)	Interpretation
1.40 + 1.40	0.53	0.34	5.31; 6.63	Strong synergy; substantial dose reduction
2.84 + 2.84	0.67	0.43	4.11; 5.46	Synergistic
5.67 + 5.67	0.83	0.42	4.01; 5.84	Synergistic at high effect level
11.34 + 11.34	0.97	0.18	8.46; 15.03	Very strong synergy
22.68 + 22.68	0.99	0.15	9.92; 19.81	Highly synergistic; maximal dose reduction

*Note:* Summary of fractional inhibition (Fa), combination index (CI), dose‐reduction index (DRI), and interaction profile for the CSE–AZE combination against *P. falciparum* 3D7. Values highlight the degree of synergism (CI < 1) and the extent of dose reduction achieved for each extract when used in combination. All were generated and presented using CompuSyn software (Version 1) as described in [17, 18].

Similarly, a high synergistic effect was observed against the DD2 lab strain with a CI of 0.614, 0.827, 0.806, 0.573 and 0.225, and DRI > 1 indicates a favourable dose reduction (Table 4).

**TABLE 4:** Synergy summary of CSE–AZE combination against *Plasmodium falciparum* DD2 lab strain.

Combination dose (CSE + AZE, μg/mL)	Fa	CI	DRI (CSE; AZE)	Interpretation
1.29 + 1.29	0.30	0.614	2.28; 5.65	Synergistic
2.58 + 2.59	0.40	0.827	1.64; 4.46	Mild synergy
5.17 + 5.17	0.60	0.806	1.60; 5.48	Synergistic
10.34 + 10.34	0.83	0.573	2.12; 9.85	Strong synergy
20.68 + 20.68	0.97	0.225	5.03; 38.21	Highly synergistic; major dose reduction

*Note:* Summary of fractional inhibition (Fa), combination index (CI), dose‐reduction index (DRI), and interaction profile for the CSE–AZE combination against *P. falciparum* DD2. Values indicate the degree of synergism (CI < 1) and the extent of dose reduction achieved for each extract when used in combination. All were generated and presented using CompuSyn software (Version 1) as described in [17, 18].

## 4. Discussion

Plants are a source of numerous novel and promising medicines [19]. They contain biologically active, naturally occurring constituents called phytochemicals, like alkaloids, glycosides, steroids, flavonoids, amino acids, saponins, tannins and more, which are responsible for their biological activities [6, 20]. Over the years, compounds isolated from herbs used traditionally for the treatment of malaria have formed the backbone of many malaria medications [21]. Phytochemical analysis of both plant samples (*C. sanguinolenta* and *A. indica*) used in this study indicates that a variety of classes of phytoconstituents were detected, most especially alkaloids. The antiplasmodial activity widely associated with *C. sanguinolenta* has been linked to the presence of alkaloids. The major alkaloid in *C. sanguinolenta* root is the indoloquinoline cryptolepine. Extensive research on this compound has shown its antiplasmodial and antipyretic effects [22, 23]. *A. indica* is known to contain some isoprenoids and nonisoprenoids, which have been responsible for the pharmacological activities reported on the plant. Azadirachtin, a known compound from neem, has demonstrated antiplasmodial activity, as well as gedunin and meldenina [24]. Numerous studies have explored the potential synergistic activity of the combination of crytolepine and other antimalarial compounds from different plants. The combination of cryptolepine with artemisinin showed a synergistic effect in both in vivo and in vitro studies against *P. berghei* NK‐65 and *P. falciparum* 3D7. More importantly, this combination did not cause acute toxicity in healthy Sprague–Dawley rats [25].

The present study (Table 2) indicates that CSE and AZE possess high antiplasmodial activity, as established by previous studies [13]. This also aligns with other scientific reports on the antiplasmodial activity of both plants [26]. This step was a required validation test before proceeding with the study’s main objective.

As established by Nortey et al. [12], most herbal antimalarial products in Ghana are made from a combination of *C. sanguinolenta* and *A. indica*. This stresses the need to scientifically evaluate how effective these herbs can be combined. The use of multiple plants in a combination does not always guarantee the desired therapeutic effect. There are herb‐herb interactions that could result in an addictive effect, a synergistic effect or even antagonism. When the interactions of the herbs result in antagonism, it could lead to toxicity, which will eventually cause harm to consumers. In this study, a computerised system (CompuSyn) was used to predict potential drug interactions. A CI value below one is considered synergism, 1 indicates additive effects, and above one is considered antagonism. The DRI also calculated how much the dosage of each drug can be reduced in combination while retaining the same efficiency. This is important for minimising systemic toxicity. These values are then used to generate isobologram graphs for the drugs being studied [27].

After the IC_50_ of both plants was established, the Compusyn software was used to quantify the extracts’ combined effects [17]. The software generates combinations of effect parameters for actual data points, summarised in Tables 3 and 4, as well as for simulated points (see the supporting information for all simulated points and curves). CSE and AZE displayed strong synergistic activity (as seen in Table 3 and Figure 4A) with CI values less than 1, and 3D7 *Plasmodium falciparum* growth was inhibited 53%–99%. Similarly, CI values for the CSE and AZE against DD2 were also less than one (Table 4 and Figure 4E). The IC50 data for the individual extracts, together with the combination results, show that CSE and AZE are more potent when used together than when used alone against both the sensitive and resistant *Plasmodium* laboratory strains (Figure 4).

CSE and AZE in combination also exerted DRI values greater than 1 (DRI > 1) in both strains (Table 3 and 3; Figure 4C and 4G). A DRI of 1 indicates that no dose reduction is achieved and the combination is simply addictive, while a DRI > 1 indicates a favourable dose reduction and DRI < 1 indicates an unfavourable reduction or antagonism. The highest DRI values were ∼19 and ∼38 in 3D7 (at 97% inhibition) and DD2 (at 99% inhibition), respectively, which means that the dose of CSE can be reduced 19‐fold and 38‐fold when used to cause > 95% inhibition in 3D7 and DD2 strains, respectively. Therefore, a lower dose of each extract leads to remarkable antiplasmodial effects in the lab strains in combination. The actual data points and simulated points were consistent for both CI and DRI determination (see supporting information for simulated data points, CompuSyn Report 1 and 2 against 3D7 and DD2, respectively) [28].

Finally, an isobologram is used to visually analyse the activity of a combination drug at doses that affect different fractions of the population. The drug activities are measured in terms of antagonism, additivity and synergism [29]. Figure 4D and 4H represent the effects of the CSE and AZE combination on the 3D7 and DD2 strains of *Plasmodium falciparum,* respectively, at 50%, 75% and 90% inhibitions. A combination data point on the diagonal line is suggestive of additivity, on the upper right suggests antagonism and on the lower right suggests synergism. CSE and AZE exerted synergy at 50%, 75% and 90% inhibitions in both strains. This isobologram data is consistent with the Fa‐CI data in Figure 4B and 4F.

## 5. Conclusion

In conclusion, *Cryptolepis sanguinolenta* and *Azadirachta indica* exerted synergistic interactions against 3D7 and DD2 strains of *Plasmodium falciparum*; hence, the rationale for their combined usage in several polyherbal antimalarial formulations. This provides a vital step in extrapolating the required dose for the combination of these plants in humans. This calls for further studies into understanding their combination mechanisms and pathways involved.

## Author Contributions

Conceptualisation, Samuel Korsah and Nathaniel Nene Djangmah Nortey; methodology, Miriam Tagoe and Felix Kwame Zoiku; formal analysis, Samuel Korsah; investigation, Joseph Kwao Amanor, Babu Jacob Yamba and Josephine Akosua Ofori‐Asamoah; writing–original draft preparation, Samuel Korsah and Nathaniel Nene Djangmah Nortey; writing–review and editing, Samuel Korsah; supervision, Samuel Korsah and project administration, Christian Akakpo and Jessica Korsah.

## Funding

This work was jointly funded by the authors with no external funding.

## Disclosure

All authors have read and agreed to the published version of the manuscript.

## Ethics Statement

The authors have nothing to report.

## Conflicts of Interest

The authors declare no conflicts of interest.

## Supporting Information

Additional supporting information can be found online in the Supporting Information section.

## Supporting information


**Supporting Information 1** RScript version.

**Supporting Information 2** CompuSyn report.

## Data Availability

The data used to support the findings of this study are included in the article and available from the corresponding author upon request.
