# Supplementary material for: Synergistic Combination of Cryptolepis sanguinolenta (Lindl.) Schlechter (Apocynaceae) and Azadirachta Indica (A. Juss) (Meliaceae) Against 3D7 and DD2 Strains of Plasmodium Falciparum
Source: Adv Pharmacol Pharm Sci. 2026 Aug 31;2026:1273876. doi: 10.1155/adpp/1273876 (PMC13527865; doi:10.1155/adpp/1273876)
Supplement: Supplementary file 1 [file ADPP-2026-1273876-s002.pdf]

CompuSyn Report 1

**Experiment Name:**	CS&AZ COMBINATION AGAINST 3D7 STRAINS
**Date:**	4/4/2024
**File Name:**	C:\Users\MAC WIN\Desktop\EFFECT CSE & AZE FINAL-report\EFFECT FINAL.cse
**Description**	COMBINATION EFFECTS OF CSE AND AZE AGAINST 3D7 STRAIN. TOP TWO MOSTLY USED PLANTS IN ANTIMALARIAL POLYHERBALS IN GHANA.

Drug: *Cryptolepine sanguinolepta* (CSE) [µg/mL]

Drug: *Azadiracta indica* (AZE) [µg/mL]

Drug Combo: Cryptolepis and Azadiracta combination (CS&AZ): (CSE+AZE); [1:1]

Data for Drug: CSE [µg/mL]

Dose	Effect
1.53	0.13
3.06	0.27
6.12	0.5
12.24	0.53
24.48	0.89

5 data points entered.

X-int: 0.83206

Y-int: -1.0922 +/- 0.18510

m: 1.31259 +/- 0.20692

Dm: 6.79298

r: 0.96469

Data for Drug: AZE [µg/mL]

Dose	Effect
1.3	0.1
2.61	0.2
5.22	0.37
10.44	0.63
20.88	0.7

5 data points entered.

X-int: 0.92329

Y-int: -1.0655 +/- 0.07735

m: 1.15401 +/- 0.09270

Dm: 8.38079

r: 0.99046

Data for Drug Combo: CS&AZ (CSE+AZE [1:1])

Dose A	Effect
1.4025+	0.53
2.835+	0.67
5.67+	0.83
11.34+	0.97
22.68+	0.99

5 data points entered.

X-int: 0.51305

Y-int: -0.8642 +/- 0.21595

m: 1.68453 +/- 0.18994

Dm: 3.25872

r: 0.98146

Dose-Effect Curve

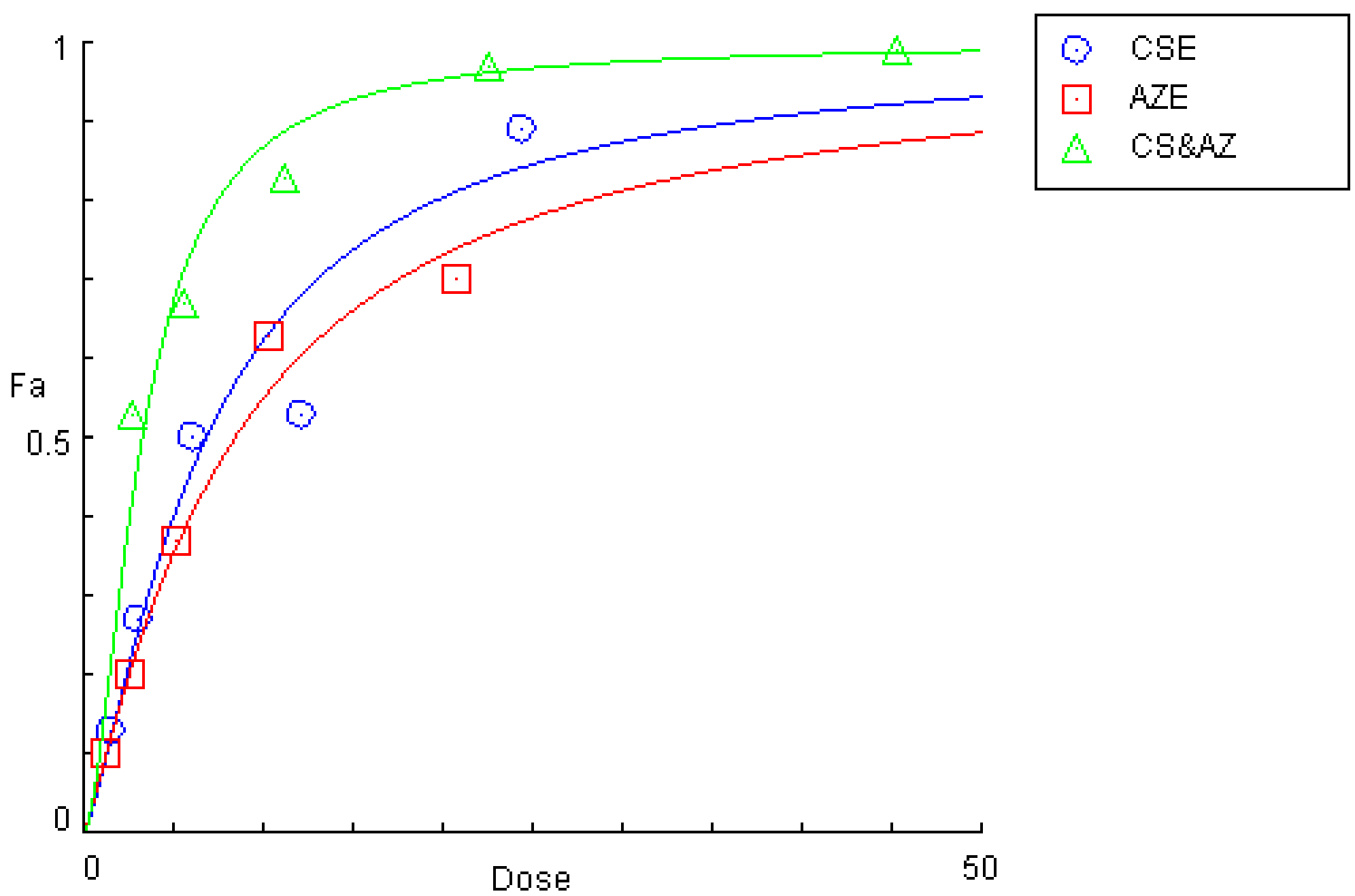

Median-Effect Plot

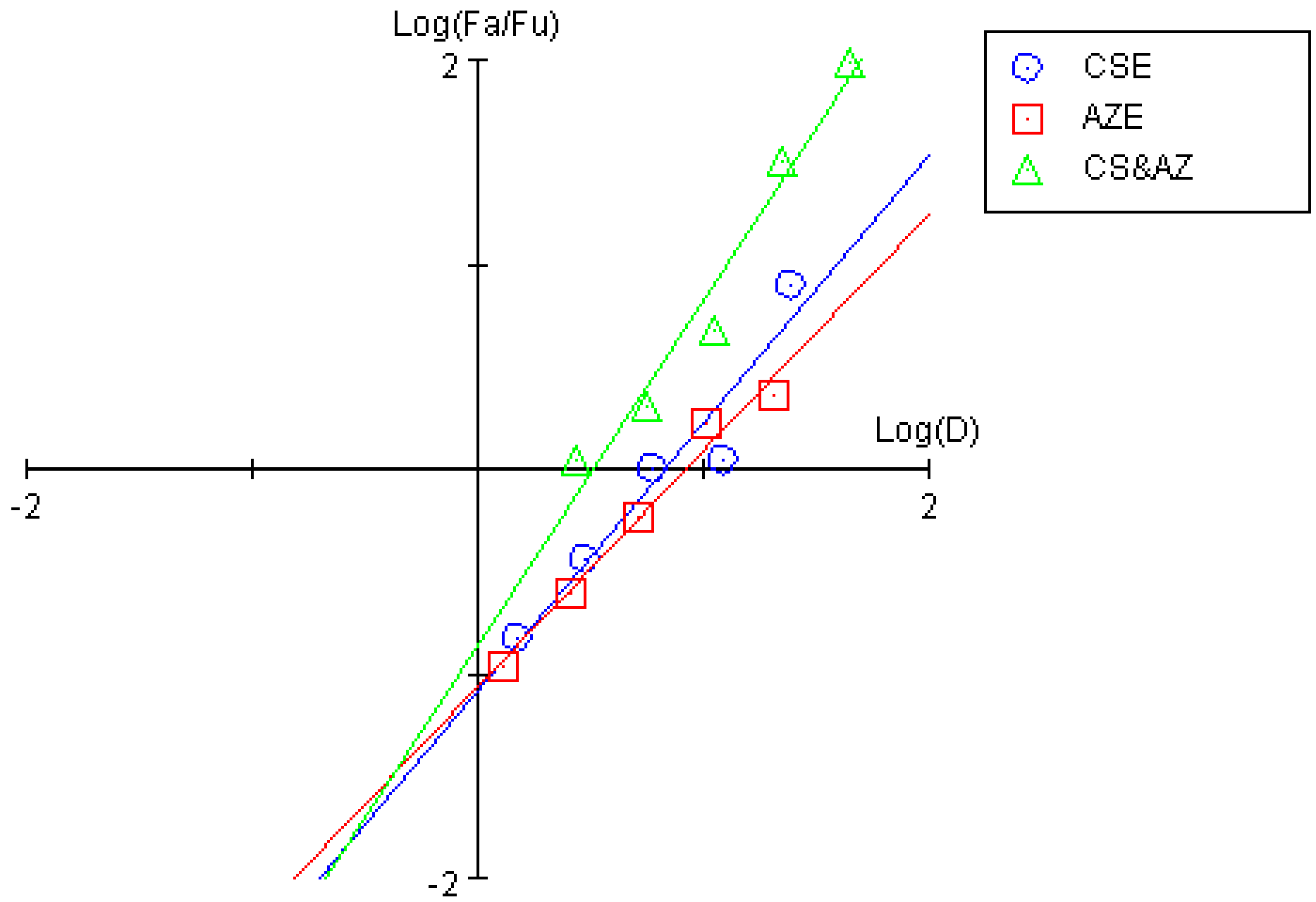

CI Data for Drug Combo: CS&AZ (CSE+AZE); [1:1]

Fa	CI Value	Total Dose
0.05	0.82784	0.56746
0.1	0.70124	0.88425
0.15	0.63325	1.16371
0.2	0.58667	1.43101
0.25	0.55093	1.69750
0.3	0.52160	1.97062
0.35	0.49638	2.25658
0.4	0.47395	2.56161
0.45	0.45345	2.89275
0.5	0.43427	3.25872
0.55	0.41595	3.67098
0.6	0.39809	4.14554
0.65	0.38033	4.70590
0.7	0.36228	5.38878
0.75	0.34344	6.25580
0.8	0.32314	7.42080
0.85	0.30026	9.12534
0.9	0.27248	12.0093
0.95	0.23321	18.7137

Fa	CI Value	Total Dose
0.97	0.20895	25.6584

CI values for actual experimental points:

Total Dose	Fa	CI Value
2.805	0.53	0.33920
5.67	0.67	0.42645
11.34	0.83	0.42062
22.68	0.97	0.18470
45.36	0.99	0.15121

Combination Index Plot

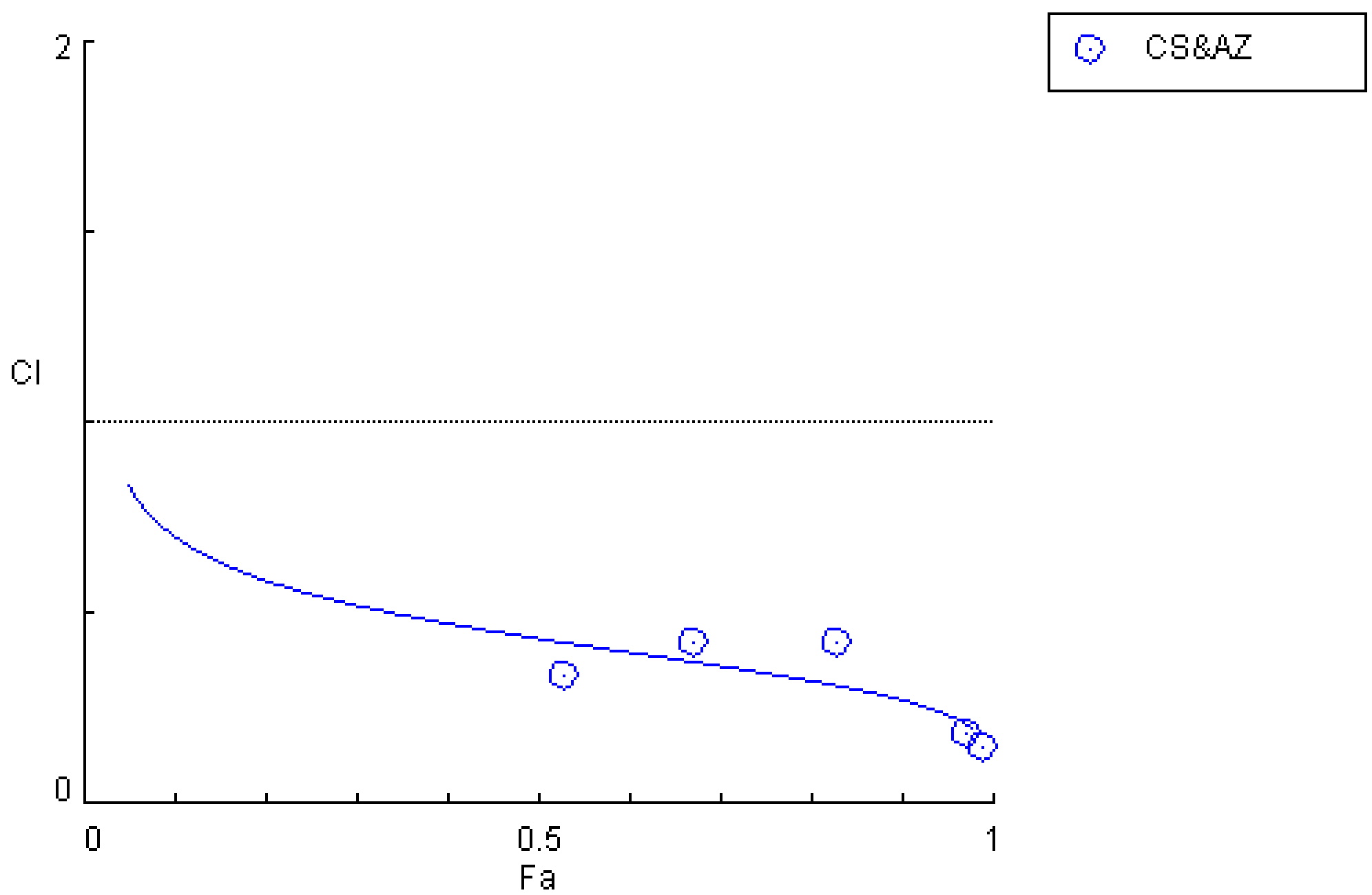

Logarithmic Combination Index Plot

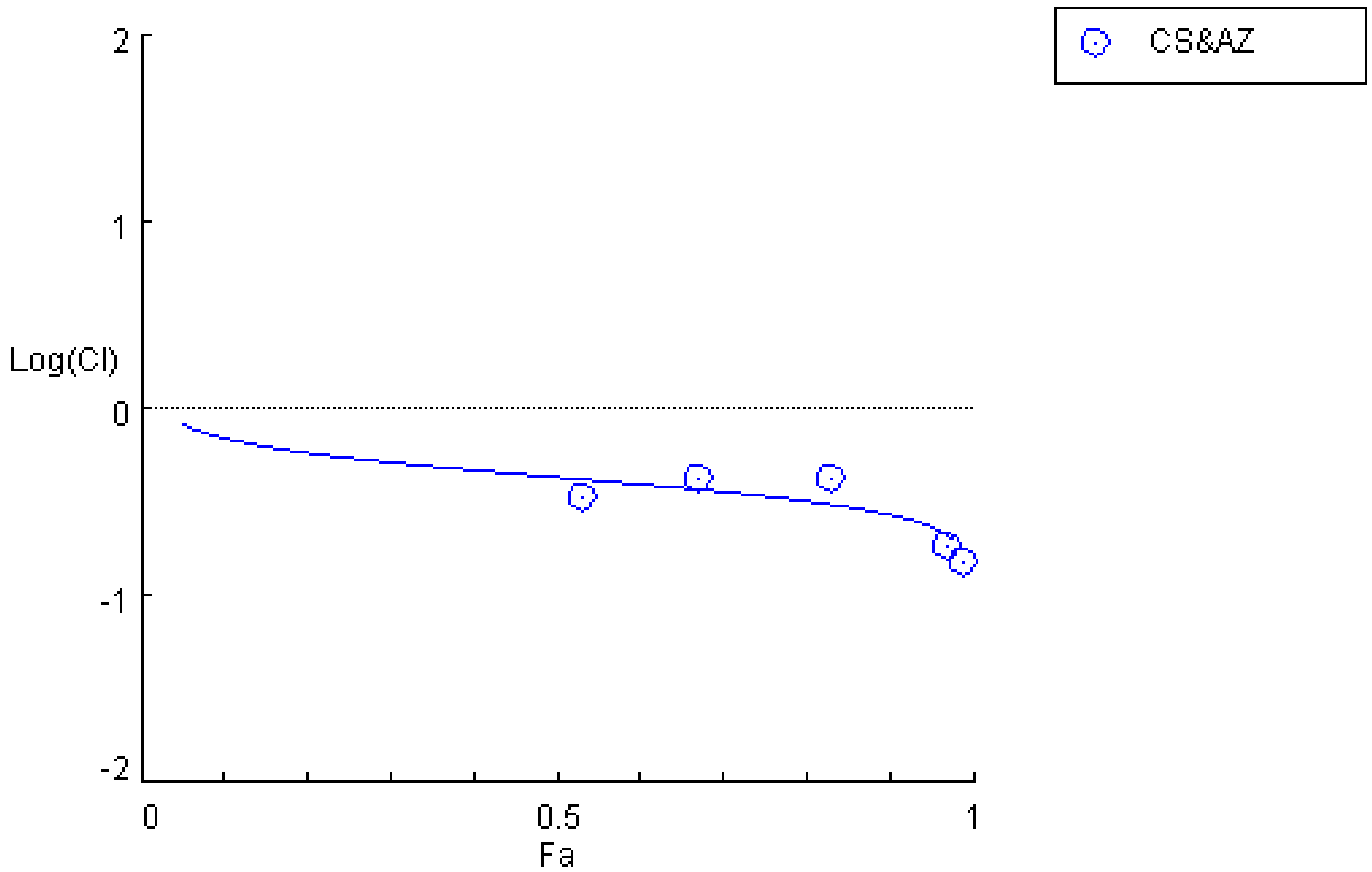

DRI Data for Drug Combo: CS&AZ (CSE+AZE [1:1])

Fa	Dose CSE	Dose AZE	DRI CSE	DRI AZE
0.05	0.72084	0.65341	2.54059	2.30294
0.1	1.27370	1.24850	2.88087	2.82387
0.15	1.81190	1.86419	3.11401	3.20388
0.2	2.36254	2.52099	3.30192	3.52338
0.25	2.94147	3.23472	3.46564	3.81116
0.3	3.56218	4.02176	3.61529	4.08172
0.35	4.23877	4.90138	3.75681	4.34408
0.4	4.98775	5.89785	3.89423	4.60480
0.45	5.82995	7.04313	4.03073	4.86950
0.5	6.79298	8.38079	4.16911	5.14362
0.55	7.91509	9.97252	4.31225	5.43316
0.6	9.25158	11.9090	4.46340	5.74547
0.65	10.8863	14.3302	4.62667	6.09031
0.7	12.9540	17.4644	4.80777	6.48177
0.75	15.6876	21.7137	5.01538	6.94193
0.8	19.5318	27.8611	5.26406	7.50892
0.85	25.4675	37.6772	5.58171	8.25772
0.9	36.2288	56.2576	6.03343	9.36898
0.95	64.0153	107.494	6.84152	11.4883

Fa	Dose CSE	Dose AZE	DRI CSE	DRI AZE
0.97	95.9827	170.400	7.48159	13.2822

DRI values calculated at experimental points

Fa	Dose CSE	Dose AZE	DRI CSE	DRI AZE
0.53	7.44410	9.30036	5.30774	6.63127
0.67	11.6514	15.4811	4.10983	5.46070
0.83	22.7350	33.1142	4.00970	5.84025
0.97	95.9827	170.400	8.46409	15.0264
0.99	225.135	449.363	9.92657	19.8132

DRI Plot for Combo: CS&AZ (CSE+AZE [1:1])

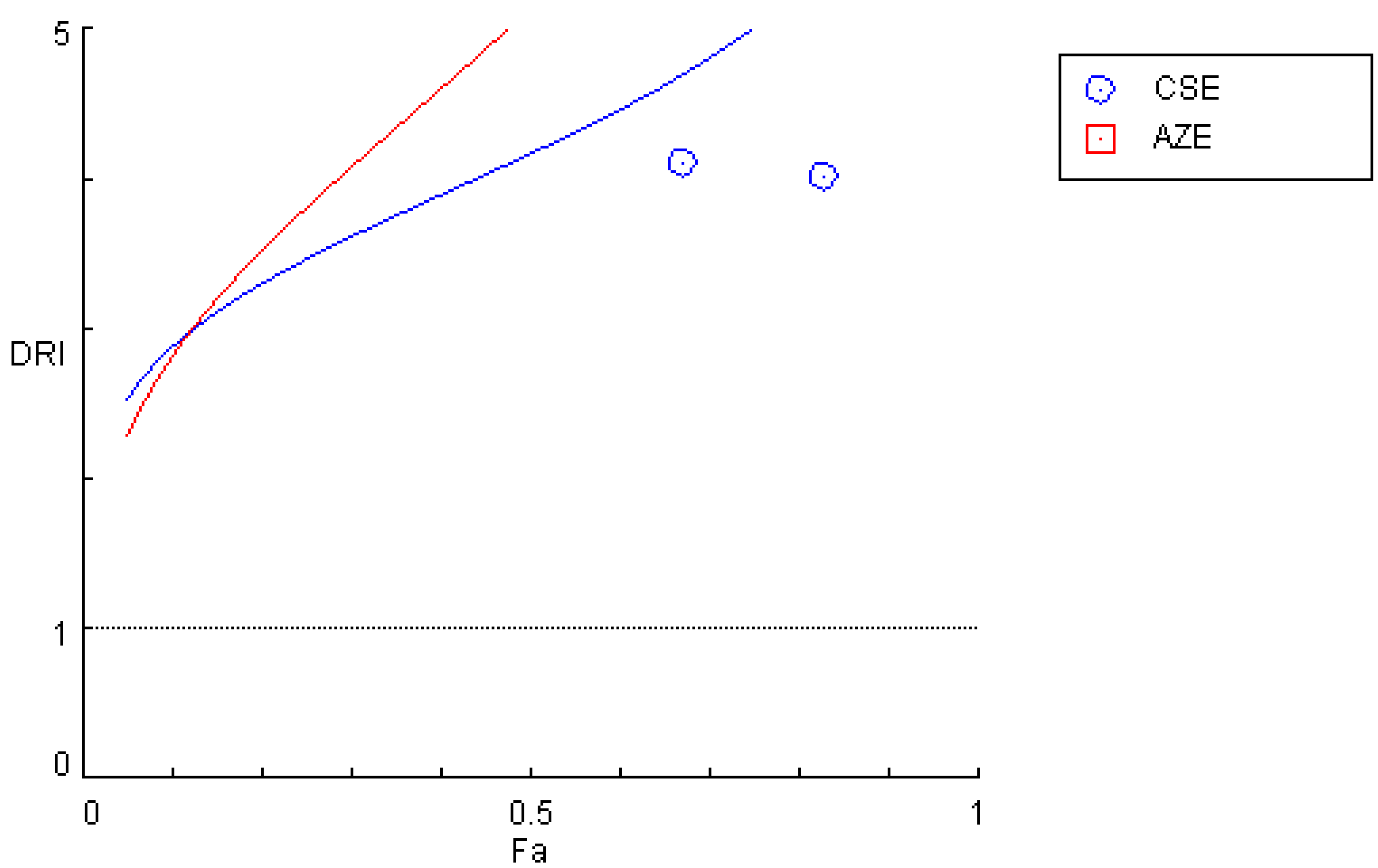

Log(DRI) Plot for Combo: CS&AZ (CSE+AZE [1:1])

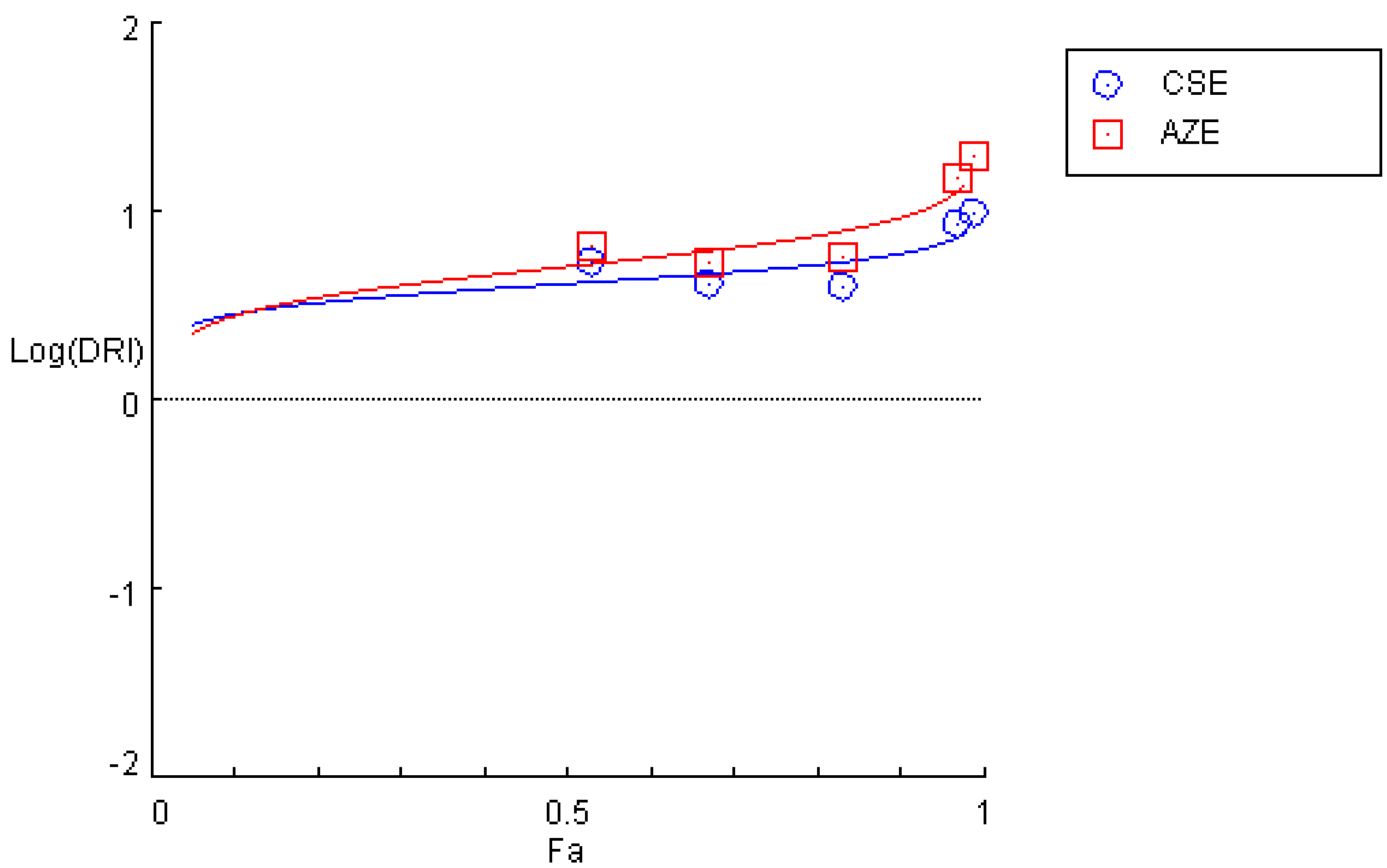

Isobologram for Combo: CS&AZ (CSE+AZE [1:1])

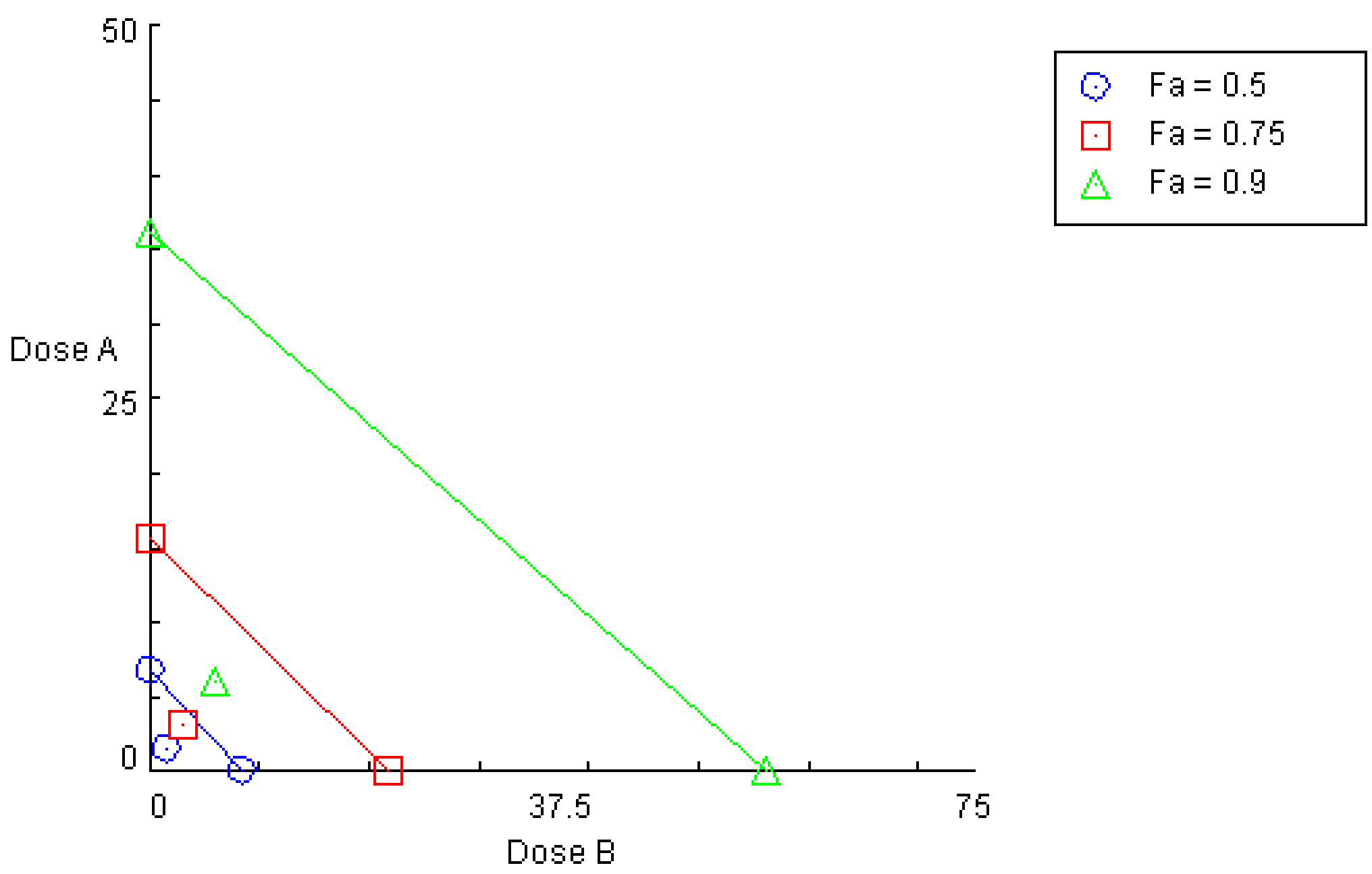

Summary Table 1

**Experiment Name:**	TWO DRUG COMBINATION
**Date:**	4/4/2024
**File Name:**	C:\Users\MAC WIN\Desktop\EFFECT CSE & AZE FINAL-report\EFFECT FINAL.cse
**Description**	COMBINATION EFFECTS OF CSE AND AZE AGAINST 3D7 STRAIN. MOST USED PLANTS IN ANTIMALARIAL POLYHERBALS IN GHANA.
**Drug:**	*Cryptolepine sanguinolepta* (CSE) [µg/mL]
**Drug:**	AZADIRACTA INDICA (AZE) [µg/mL]
**Drug Combo:**	CRYPTOLEPIS AND AZADIRACTA COMBINATION (CS&AZ) (CSE+AZE [1:1])

Drug/Combo	Dm	m	r
CSE	6.79298	1.31259	0.96469
AZE	8.38079	1.15401	0.99046
CS&AZ	3.25872	1.68453	0.98146

CI values at:

Combo	ED50	ED75	ED90	ED95
CS&AZ	0.43427	0.34344	0.27248	0.23321

Data for Fa = 0.5

Drug/Combo	CI value	Dose CSE	Dose AZE
CSE		6.79298	
AZE			8.38079
CS&AZ	0.43427	1.62936	1.62936

Data for Fa = 0.75

Drug/Combo	CI value	Dose CSE	Dose AZE
CSE		15.6876	
AZE			21.7137
CS&AZ	0.34344	3.12790	3.12790

Data for Fa = 0.9

Drug/Combo	CI value	Dose CSE	Dose AZE
CSE		36.2288	
AZE			56.2576
CS&AZ	0.27248	6.00467	6.00467

Data for Fa = 0.95

Drug/Combo	CI value	Dose CSE	Dose AZE
CSE		64.0153	
AZE			107.494
CS&AZ	0.23321	9.35687	9.35687

Data for Fa = 0.97

Drug/Combo	CI value	Dose CSE	Dose AZE
CSE		95.9827	
AZE			170.400
CS&AZ	0.20895	12.8292	12.8292

CompuSyn Report 2

**Experiment Name:**	CS&AZ COMBINATION AGAINT DD2
**Date:**	5/4/24
**File Name:**	C:\Users\MAC WIN\Desktop\EFFECT CSE & AZE FINAL-report\DD2 1.cse
**Description**	COMBINATION EFFECTS OF CSE AND AZE AGAINST 3D7 STRAIN. TOP TWO MOSTLY USED PLANTS IN ANTIMALARIAL POLYHERBALS IN GHANA.
**Drug:**	CRYPTOLEPIS SANQUINOLEPTA (CSE) [μg/mL]
**Drug:**	AZADIRACTA INDICA (AZE) [μg/mL]
**Drug Combo:**	CRYPTOLEPIS AND AZADIRACTA (CS&AZ) (CSE+AZE); [1:1]

Data for Drug: CSE [μg/mL]

Dose	Effect
1.28	0.13
2.56	0.3
5.12	0.37
10.24	0.73
20.48	0.8

5 data points entered.

X-int: 0.77362

Y-int: -0.9394 +/- 0.11539

m: 1.21423 +/- 0.13949

Dm: 5.93771

r: 0.98077

Data for Drug: AZE [µg/mL]

Dose	Effect
1.305	0.1
2.61	0.13
5.22	0.2
10.44	0.33
20.88	0.6

5 data points entered.

**X-int:**	1.26224
**Y-int:**	-1.1651 +/- 0.10705
**m:**	0.92306 +/- 0.12829
**Dm:**	18.2911
**r:**	0.97223

Data for Drug Combo: CS&AZ (CSE+AZE); [1:1]

Dose A	Effect
1.2925+	0.3
2.585+	0.4
5.17+	0.6
10.34+	0.83
20.68+	0.97

5 data points entered.

**X-int:**	0.77600
**Y-int:**	-1.1909 +/- 0.25519
**m:**	1.53472 +/- 0.23195
**Dm:**	5.97037
**r:**	0.96740

Dose-Effect Curve

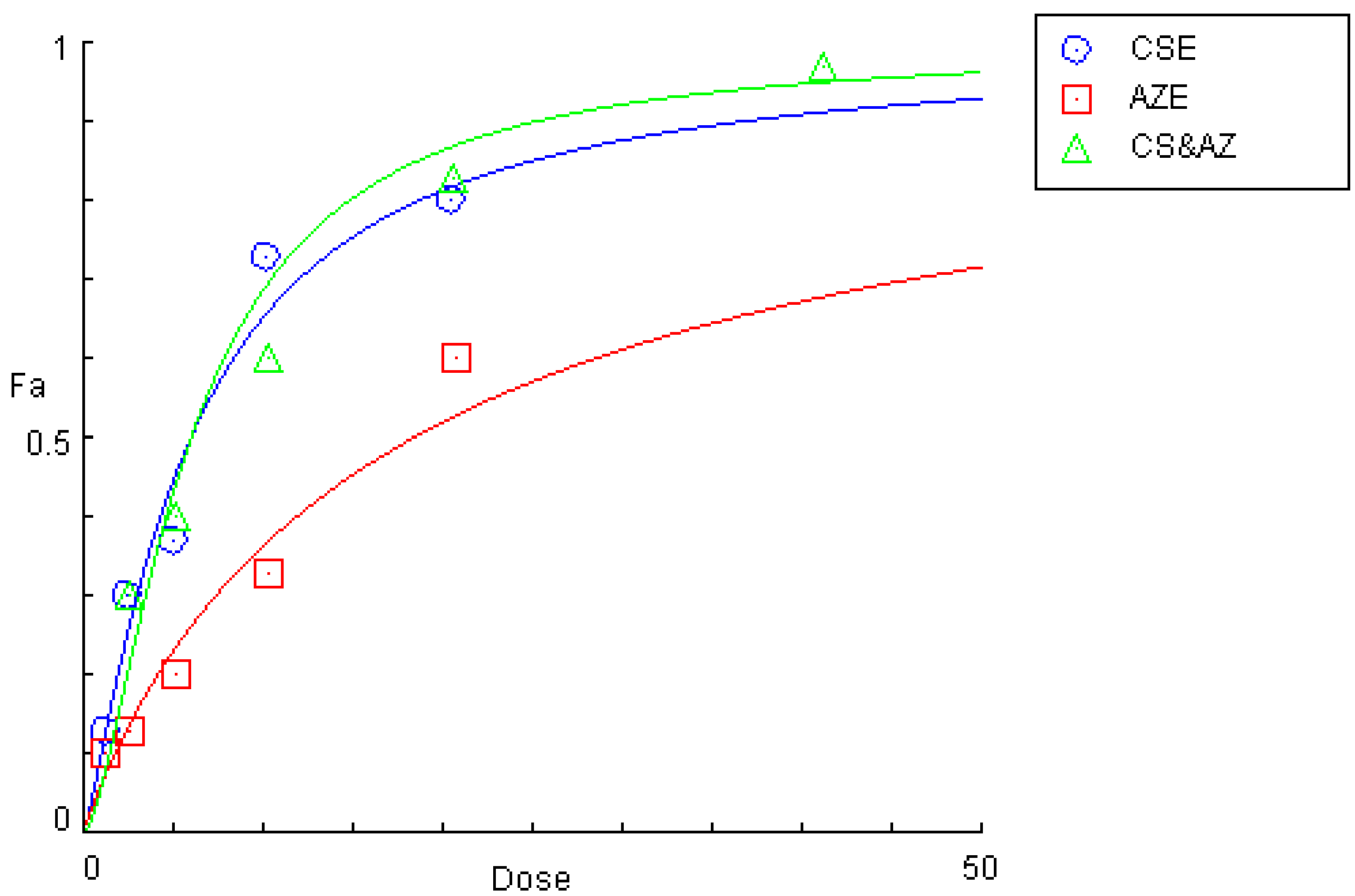

Median-Effect Plot

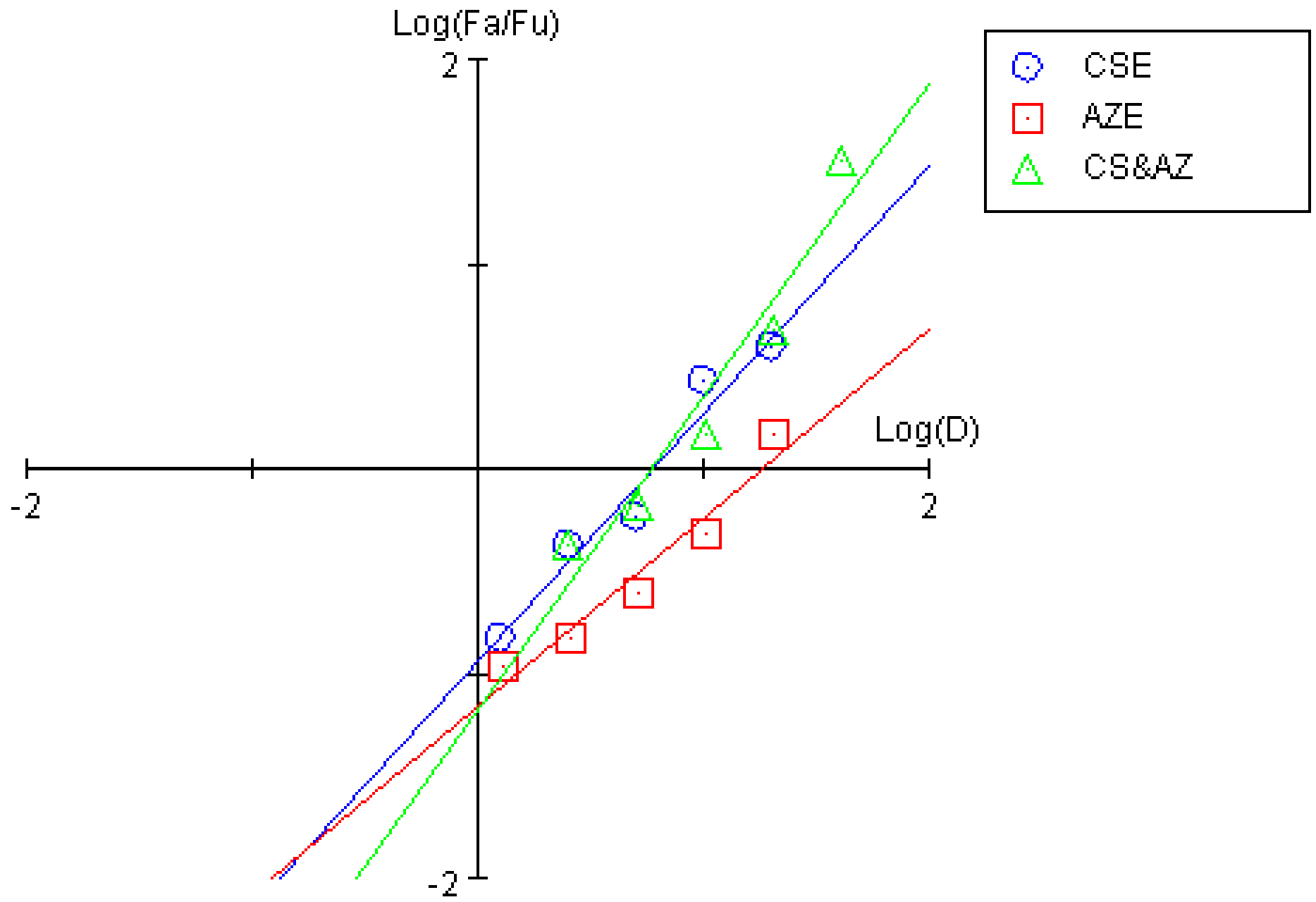

CI Data for Drug Combo: CS&AZ (CSE+AZE); [1:1]

Fa	CI Value	Total Dose
0.05	1.41612	0.87657
0.1	1.15506	1.42637

Fa	CI Value	Total Dose
0.15	1.02264	1.92817
0.2	0.93506	2.41941
0.25	0.86957	2.91821
0.3	0.81691	3.43743
0.35	0.77244	3.98865
0.4	0.73349	4.58420
0.45	0.69838	5.23861
0.5	0.66595	5.97037
0.55	0.63535	6.80436
0.6	0.60588	7.77570
0.65	0.57690	8.93669
0.7	0.54778	10.3698
0.75	0.51776	12.2148
0.8	0.48580	14.7331
0.85	0.45025	18.4866
0.9	0.40774	24.9902
0.95	0.34876	40.6646
0.97	0.31290	57.4996

CI values for actual experimental points:

Total Dose	Fa	CI Value
2.585	0.3	0.61433
5.17	0.4	0.82722
10.34	0.6	0.80569
20.68	0.83	0.57326
41.36	0.97	0.22507

Combination Index Plot

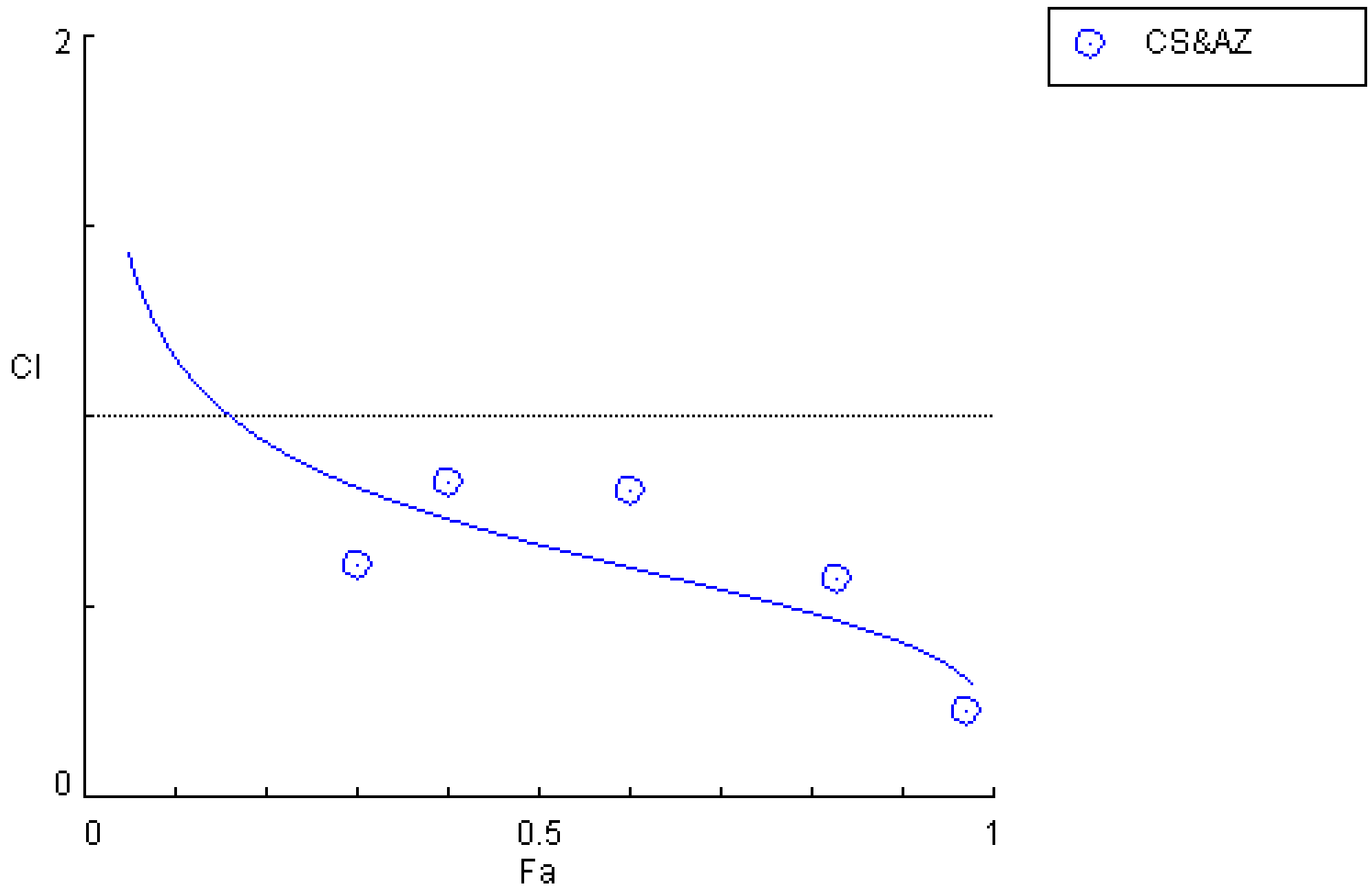

Logarithmic Combination Index Plot

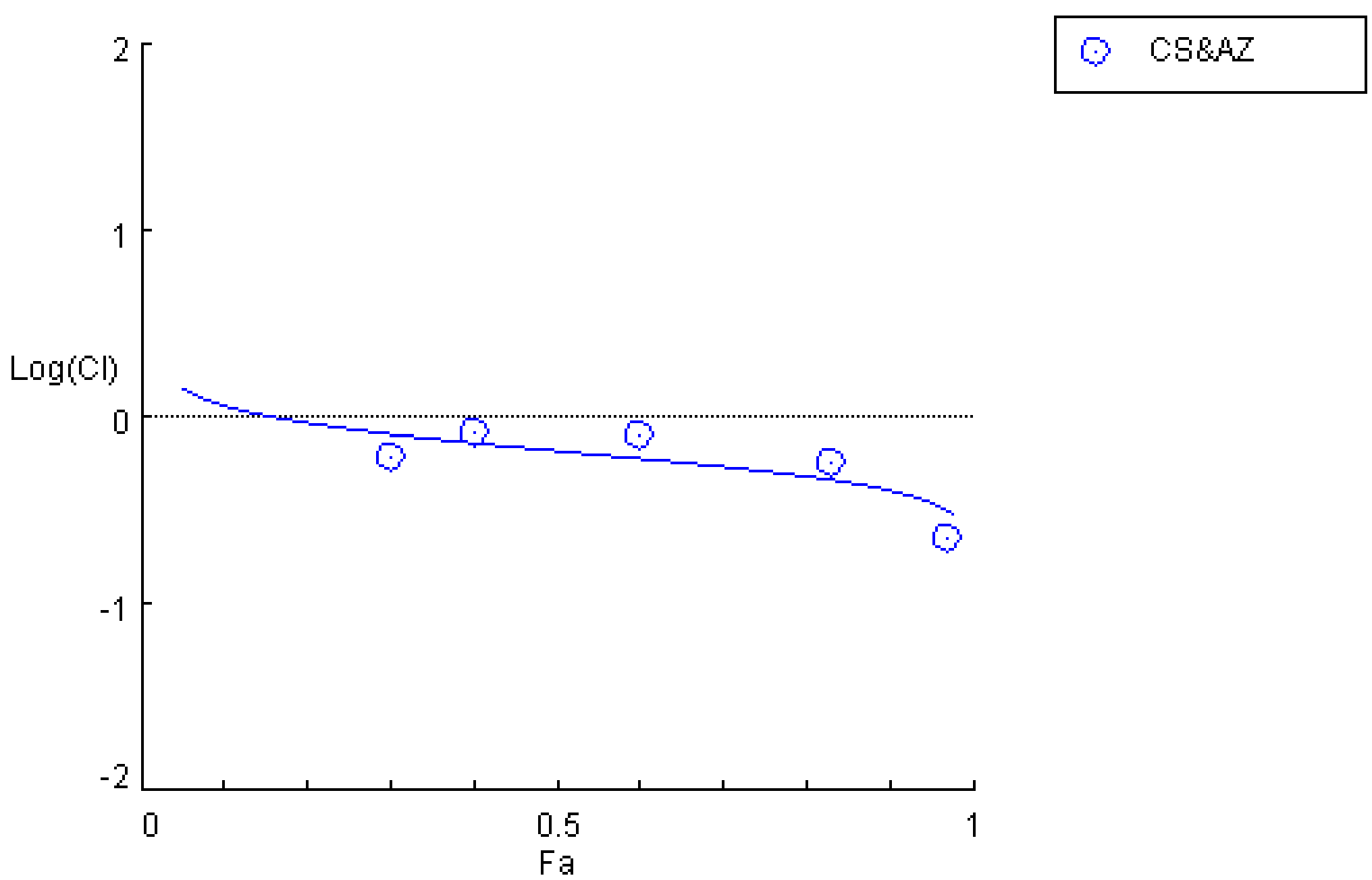

DRI Data for Drug Combo: CS&AZ (CSE+AZE); [1:1]

Fa	Dose CSE	Dose AZE	DRI CSE	DRI AZE
0.05	0.52539	0.75318	1.19874	1.71846
0.1	0.97216	1.69222	1.36312	2.37276

Fa	Dose CSE	Dose AZE	DRI CSE	DRI AZE
0.15	1.42300	2.79331	1.47601	2.89737
0.2	1.89576	4.07376	1.56713	3.36757
0.25	2.40258	5.56350	1.64661	3.81295
0.3	2.95506	7.30450	1.71934	4.24998
0.35	3.56621	9.35374	1.78818	4.69017
0.4	4.25203	11.7888	1.85508	5.14325
0.45	5.03321	14.7172	1.92158	5.61875
0.5	5.93771	18.2911	1.98906	6.12729
0.55	7.00475	22.7329	2.05890	6.68186
0.6	8.29166	28.3798	2.13271	7.29961
0.65	9.88622	35.7680	2.21250	8.00476
0.7	11.9309	45.8026	2.30108	8.83386
0.75	14.6744	60.1356	2.40272	9.84637
0.8	18.5975	82.1268	2.52459	11.1486
0.85	24.7761	119.774	2.68044	12.9579
0.9	36.2660	197.708	2.90241	15.8228
0.95	67.1050	444.206	3.30042	21.8473
0.97	103.971	790.183	3.61642	27.4848

DRI values calculated at experimental points

Fa	Dose CSE	Dose AZE	DRI CSE	DRI AZE
0.3	2.95506	7.30450	2.28631	5.65145
0.4	4.25203	11.7888	1.64489	4.56048
0.6	8.29166	28.3798	1.60380	5.48933
0.83	21.9154	101.923	2.11948	9.85711
0.97	103.971	790.183	5.02763	38.2100

DRI Plot for Combo: CS&AZ (CSE+AZE); [1:1]

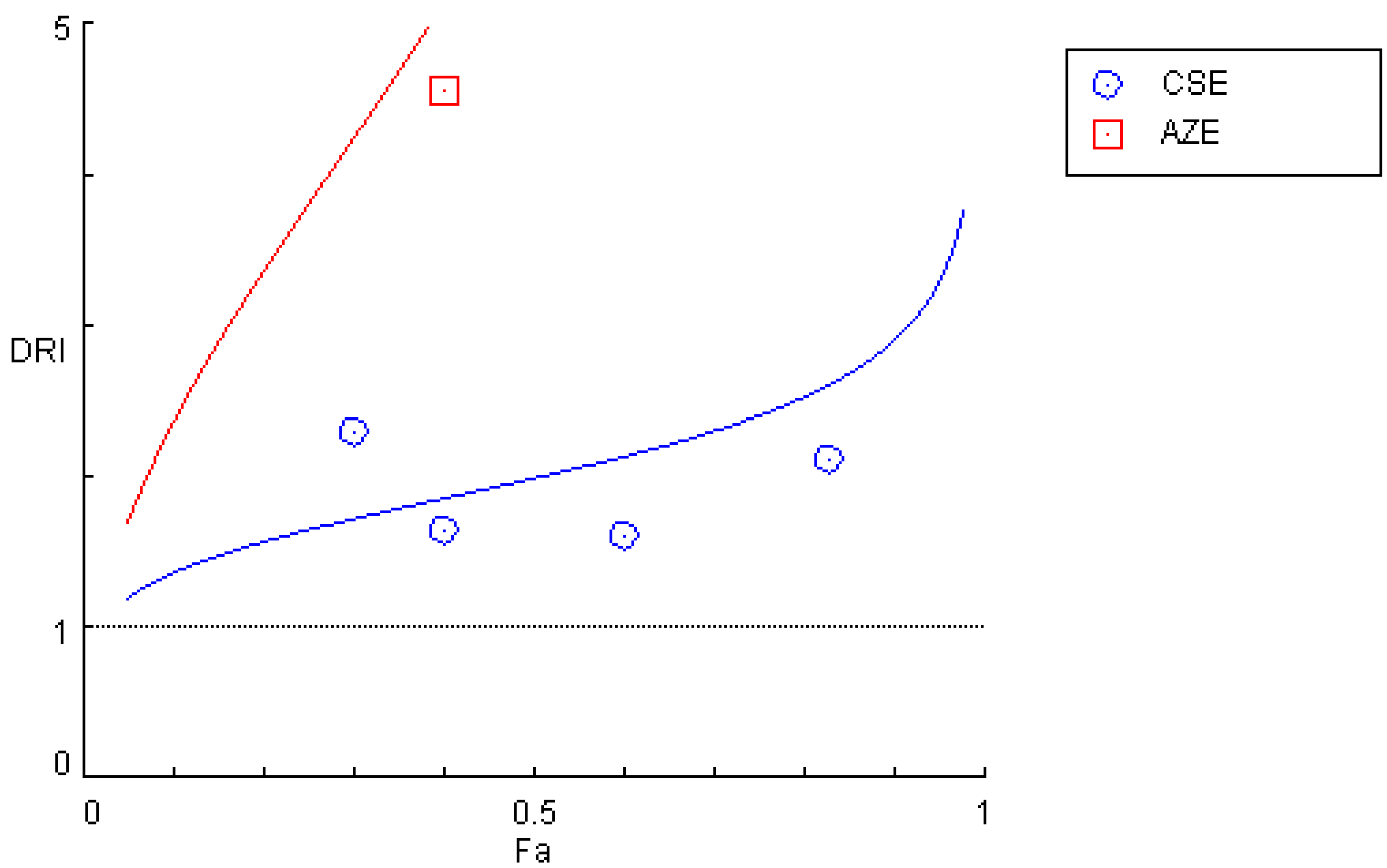

Log(DRI) Plot for Combo: CS&AZ (CSE+AZE); [1:1]

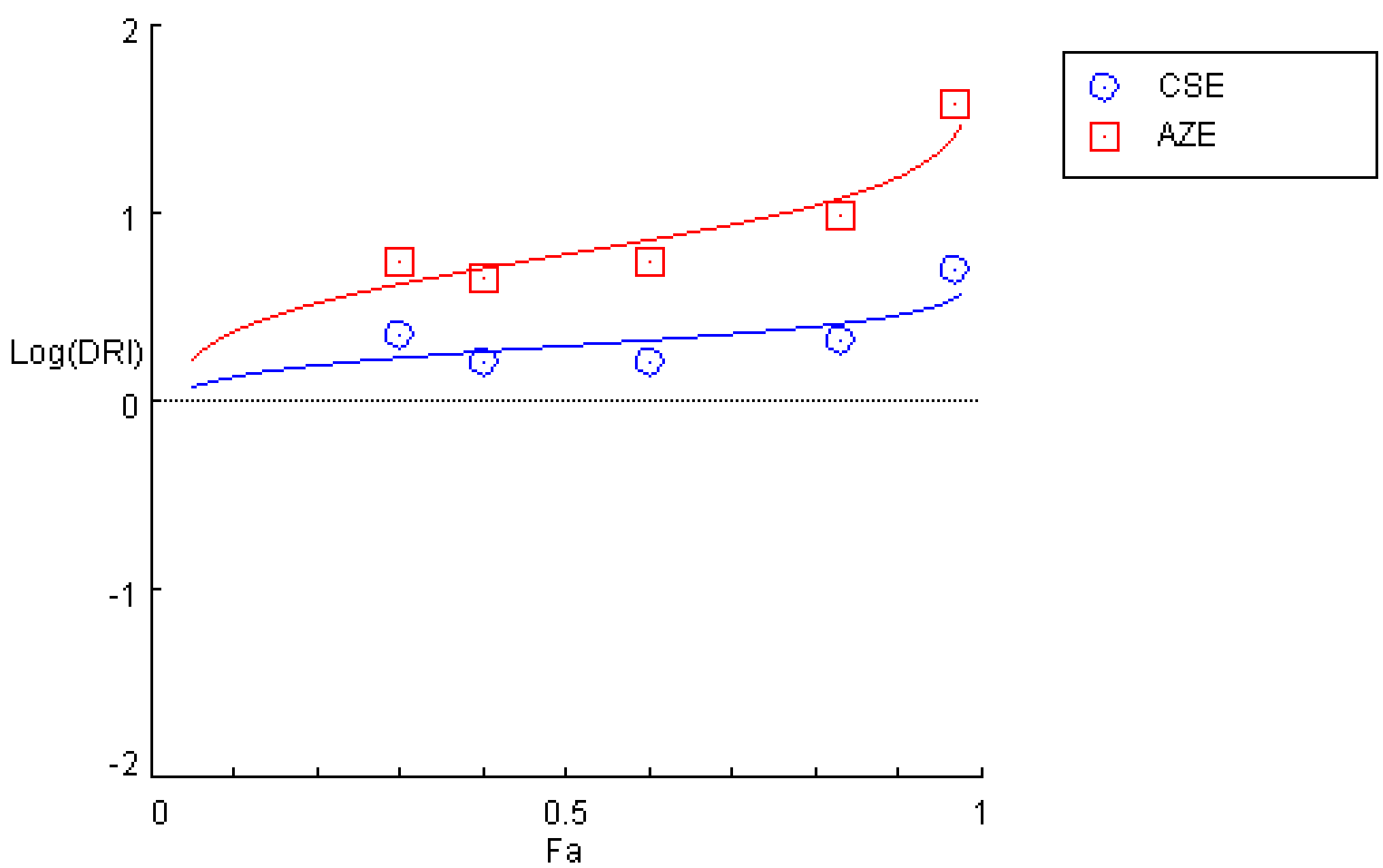

Isobologram for Combo: CS&AZ (CSE+AZE); [1:1]

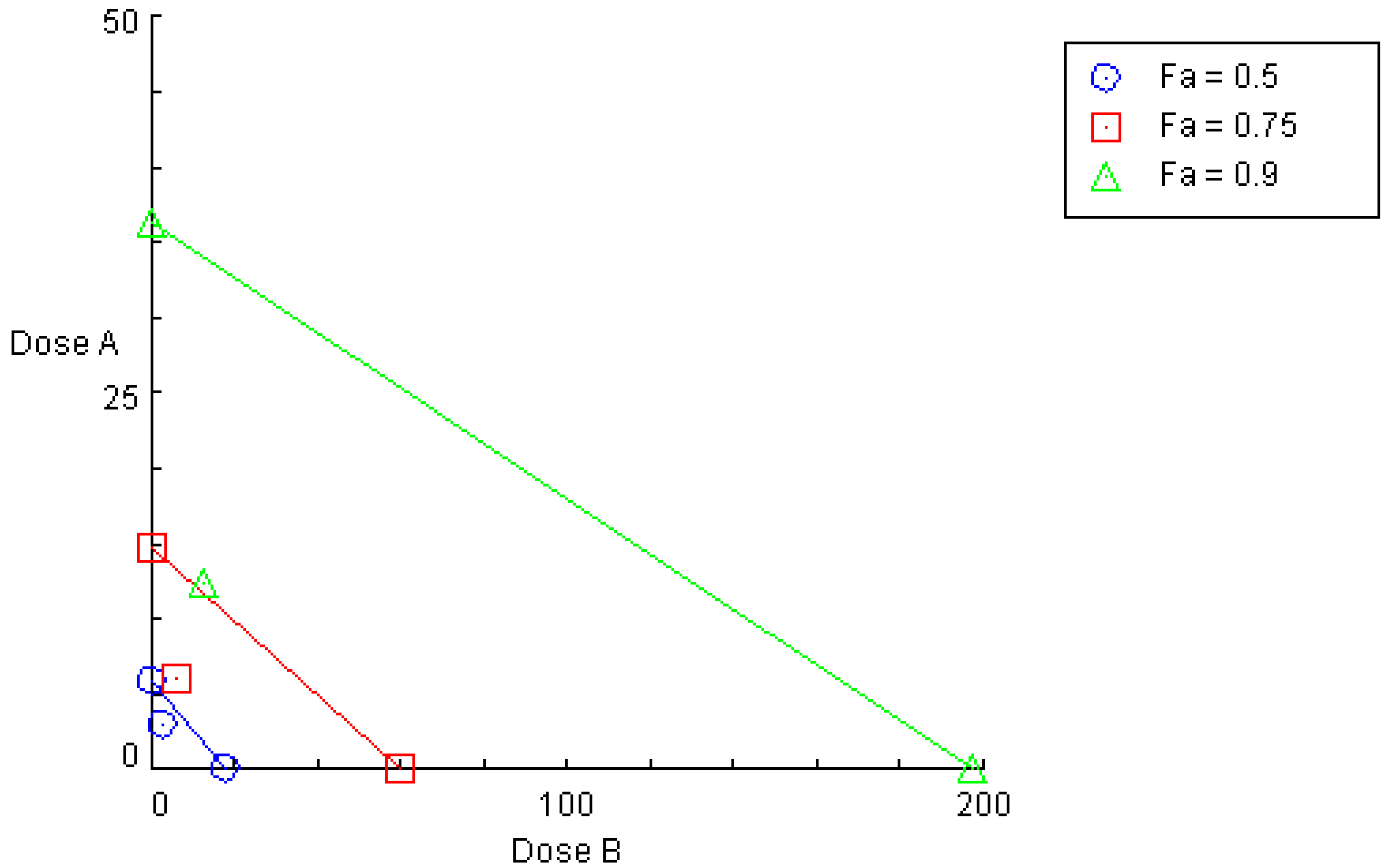

Summary Table 2

**Experiment Name:**	COMBINATION CSE AZE AGAINT DD2
**Date:**	5/4/24
**File Name:**	C:\Users\MAC WIN\Desktop\EFFECT CSE & AZE FINAL-report\DD2 1.cse
**Description**	ANTIMALARIAL ACTIVITY OF TWO extracts
**Drug:**	CRYPTOLEPIS SANQUINOLEPTA (CSE) [µg/mL]
**Drug:**	AZADIRACTA INDICA (AZE) [µg/mL]
**Drug Combo:**	CRYPTOLEPIS AND AZADIRACTA (CS&AZ) (CSE+AZE); [1:1]

**Drug/Combo**	**Dm**	**m**	**r**
CSE	5.93771	1.21423	0.98077
AZE	18.2911	0.92306	0.97223
CS&AZ	5.97037	1.53472	0.96740

CI values at:

**Combo**	**ED50**	**ED75**	**ED90**	**ED95**
CS&AZ	0.66595	0.51776	0.40774	0.34876

Data for Fa = 0.5

Drug/Combo	CI value	Dose CSE	Dose AZE
CSE		5.93771	
AZE			18.2911
CS&AZ	0.66595	2.98519	2.98519

Data for Fa = 0.75

Drug/Combo	CI value	Dose CSE	Dose AZE
CSE		14.6744	
AZE			60.1356
CS&AZ	0.51776	6.10739	6.10739

Data for Fa = 0.9

Drug/Combo	CI value	Dose CSE	Dose AZE
CSE		36.2660	
AZE			197.708
CS&AZ	0.40774	12.4951	12.4951

Data for Fa = 0.95

Drug/Combo	CI value	Dose CSE	Dose AZE
CSE		67.1050	
AZE			444.206
CS&AZ	0.34876	20.3323	20.3323

Data for Fa = 0.97

Drug/Combo	CI value	Dose CSE	Dose AZE
CSE		103.971	
AZE			790.183
CS&AZ	0.31290	28.7498	28.7498
