# Supplementary material for: Synergistic Combination of Cryptolepis sanguinolenta (Lindl.) Schlechter (Apocynaceae) and Azadirachta Indica (A. Juss) (Meliaceae) Against 3D7 and DD2 Strains of Plasmodium Falciparum
Source: Adv Pharmacol Pharm Sci. 2026 Aug 31;2026:1273876. doi: 10.1155/adpp/1273876 (PMC13527865; doi:10.1155/adpp/1273876)
Supplement: Supplementary file 2 [file ADPP-2026-1273876-s001.docx]

***Note- This script uses ggplot2 to replot CompuSyn data for both 3D7 and DD2 for :***

1. **Dose–Effect Curve**
2. **Median‑Effect Plot**
3. **Combination Index (CI) Plot**
4. **Isobologram**

**R SCRIPT —COMPUSYN PLOTS (3D7 + DD2)**

**# ============================**

**# REQUIRED PACKAGES**

**# ============================**

**library(ggplot2)**

**library(patchwork)**

**theme_nature <- function() {**

**theme_bw(base_size = 10) +**

**theme(**

**panel.grid = element_blank(),**

**panel.border = element_blank(),**

**axis.line = element_line(color="black", linewidth=0.5),**

**axis.ticks = element_line(color="black", linewidth=0.5),**

**axis.text = element_text(color="black"),**

**plot.title = element_text(face="bold", hjust=0)**

**)**

**}**

**# ============================**

**# RAW DATA (FROM YOUR REPORTS)**

**# ============================**

**## 3D7**

**dose_3d7_cse <- c(1.53, 3.06, 6.12, 12.24, 24.48)**

**fa_3d7_cse	<- c(0.13, 0.27, 0.50, 0.53, 0.89)**

**dose_3d7_aze <- c(1.3, 2.61, 5.22, 10.44, 20.88)**

**fa_3d7_aze	<- c(0.10, 0.20, 0.37, 0.63, 0.70)**

**dose_3d7_combo <- c(1.4025, 2.835, 5.67, 11.34, 22.68)**

**fa_3d7_combo	<- c(0.53, 0.67, 0.83, 0.97, 0.99)**

**Dm_3d7_cse <- 6.79298; m_3d7_cse <- 1.31259**

**Dm_3d7_aze <- 8.38079; m_3d7_aze <- 1.15401**

**Dm_3d7_combo <- 3.25872; m_3d7_combo <- 1.68453**

**fa_3d7_ci <- c(0.53, 0.67, 0.83, 0.97, 0.99)**

**ci_3d7	<- c(0.33920, 0.42645, 0.42062, 0.18470, 0.15121)**

**iso_3d7_cse <- c(1.62936, 3.12790, 6.00467)**

**iso_3d7_aze <- c(1.62936, 3.12790, 6.00467)**

**## DD2**

**dose_dd2_cse <- c(1.28, 2.56, 5.12, 10.24, 20.48)**

**fa_dd2_cse	<- c(0.13, 0.30, 0.37, 0.73, 0.80)**

**dose_dd2_aze <- c(1.305, 2.61, 5.22, 10.44, 20.88)**

**fa_dd2_aze	<- c(0.10, 0.13, 0.20, 0.33, 0.60)**

**dose_dd2_combo <- c(1.2925, 2.585, 5.17, 10.34, 20.68)**

**fa_dd2_combo	<- c(0.30, 0.40, 0.60, 0.83, 0.97)**

**Dm_dd2_cse <- 5.93771; m_dd2_cse <- 1.21423**

**Dm_dd2_aze <- 18.2911; m_dd2_aze <- 0.92306**

**Dm_dd2_combo <- 5.97037; m_dd2_combo <- 1.53472**

**fa_dd2_ci <- c(0.3, 0.4, 0.6, 0.83, 0.97)**

**ci_dd2	<- c(0.61433, 0.82722, 0.80569, 0.57326, 0.22507)**

**iso_dd2_cse <- c(2.98519, 6.10739, 12.4951)**

**iso_dd2_aze <- c(2.98519, 6.10739, 12.4951)**

**# ============================**

**# MEDIAN-EFFECT FIT FUNCTION**

**# ============================**

**median_effect_curve <- function(Dm, m, doses) {**

**(doses^m) / (doses^m + Dm^m)**

**}**

**# ============================**

**# 1. DOSE–EFFECT CURVES**

**# ============================**

**dseq <- 10^seq(-1, 2, length.out = 200)**

**plot_dose_effect <- function(dose_cse, fa_cse, dose_aze, fa_aze,**

**dose_combo, fa_combo, Dm_cse, m_cse,**

**Dm_aze, m_aze, Dm_combo, m_combo, title) {**

**df <- data.frame(**

**dose = c(dseq, dseq, dseq, dose_cse, dose_aze, dose_combo),**

**fa	= c(**

**median_effect_curve(Dm_cse, m_cse, dseq),**

**median_effect_curve(Dm_aze, m_aze, dseq),**

**median_effect_curve(Dm_combo, m_combo, dseq),**

**fa_cse, fa_aze, fa_combo**

**),**

**type = factor(c(**

**rep("CSE_fit", length(dseq)),**

**rep("AZE_fit", length(dseq)),**

**rep("Combo_fit", length(dseq)),**

**rep("CSE", length(dose_cse)),**

**rep("AZE", length(dose_aze)),**

**rep("Combo", length(dose_combo))**

**))**

**)**

**ggplot(df, aes(dose, fa)) +**

**geom_line(data=subset(df, grepl("fit", type)),**

**aes(color=type), linewidth=0.7) +**

**geom_point(data=subset(df, !grepl("fit", type)),**

**aes(color=type), size=2) +**

**scale_x_log10() +**

**scale_color_manual(values=c(**

**"CSE_fit"="blue", "AZE_fit"="red", "Combo_fit"="green",**

**"CSE"="blue", "AZE"="red", "Combo"="green"**

**)) +**

**labs(title=title, x="Dose (µg/mL)", y="Fa") +**

**theme_nature()**

**}**

**pA_3d7 <- plot_dose_effect(**

**dose_3d7_cse, fa_3d7_cse,**

**dose_3d7_aze, fa_3d7_aze,**

**dose_3d7_combo, fa_3d7_combo,**

**Dm_3d7_cse, m_3d7_cse,**

**Dm_3d7_aze, m_3d7_aze,**

**Dm_3d7_combo, m_3d7_combo,**

**"3D7 Dose–Effect"**

**)**

**pA_dd2 <- plot_dose_effect(**

**dose_dd2_cse, fa_dd2_cse,**

**dose_dd2_aze, fa_dd2_aze,**

**dose_dd2_combo, fa_dd2_combo,**

**Dm_dd2_cse, m_dd2_cse,**

**Dm_dd2_aze, m_dd2_aze,**

**Dm_dd2_combo, m_dd2_combo,**

**"DD2 Dose–Effect"**

**)**

**# ============================**

**# 2. MEDIAN-EFFECT PLOTS**

**# ============================**

**plot_median_effect <- function(dose, fa, title) {**

**fu <- 1 - fa**

**df <- data.frame(**

**logD = log10(dose),**

**logFaFu = log10(fa / fu)**

**)**

**ggplot(df, aes(logD, logFaFu)) +**

**geom_point(color="black", size=2) +**

**labs(title=title, x="log10(Dose)", y="log10(Fa/Fu)") +**

**theme_nature()**

**}**

**pB_3d7 <- plot_median_effect(dose_3d7_cse, fa_3d7_cse, "3D7 Median‑Effect")**

**pB_dd2 <- plot_median_effect(dose_dd2_cse, fa_dd2_cse, "DD2 Median‑Effect")**

**# ============================**

**# 3. CI PLOTS**

**# ============================**

**plot_ci <- function(fa, ci, title) {**

**df <- data.frame(Fa=fa, CI=ci)**

**ggplot(df, aes(Fa, CI)) +**

**geom_line(color="black", linewidth=0.7) +**

**geom_point(color="black", size=2) +**

**geom_hline(yintercept=1, linetype="dashed", color="grey40") +**

**labs(title=title, x="Fa", y="CI") +**

**theme_nature()**

**}**

**pC_3d7 <- plot_ci(fa_3d7_ci, ci_3d7, "3D7 CI Plot")**

**pC_dd2 <- plot_ci(fa_dd2_ci, ci_dd2, "DD2 CI Plot")**

**# ============================**

**# 4. ISOBLOGRAMS**

**# ============================**

**plot_iso <- function(cse, aze, title) {**

**df <- data.frame(CSE=cse, AZE=aze)**

**ggplot(df, aes(CSE, AZE)) +**

**geom_line(color="black", linewidth=0.7) +**

**geom_point(color="black", size=2) +**

**labs(title=title, x="Dose CSE", y="Dose AZE") +**

**theme_nature()**

**}**

**pD_3d7 <- plot_iso(iso_3d7_cse, iso_3d7_aze, "3D7 Isobologram")**

**pD_dd2 <- plot_iso(iso_dd2_cse, iso_dd2_aze, "DD2 Isobologram")**

**# ============================**

**# SAVE ALL 8 PLOTS**

**# ============================**

**ggsave("3D7_DoseEffect.png", pA_3d7, width=4, height=4, dpi=600)**

**ggsave("3D7_MedianEffect.png", pB_3d7, width=4, height=4, dpi=600)**

**ggsave("3D7_CI.png", pC_3d7, width=4, height=4, dpi=600)**

**ggsave("3D7_Isobologram.png", pD_3d7, width=4, height=4, dpi=600)**

**ggsave("DD2_DoseEffect.png", pA_dd2, width=4, height=4, dpi=600)**

**ggsave("DD2_MedianEffect.png", pB_dd2, width=4, height=4, dpi=600)**

**ggsave("DD2_CI.png", pC_dd2, width=4, height=4, dpi=600)**

**ggsave("DD2_Isobologram.png", pD_dd2, width=4, height=4, dpi=600)**

**R SCRIPT — Combine the 4 Plots Into One Figure (3D7 + DD2)**

library(ggplot2)

library(patchwork)

\# -------------------------

\# Nature-style theme

\# -------------------------

theme_nature <- function() {

theme_bw(base_size = 10) +

theme(

panel.grid = element_blank(),

panel.border = element_blank(),

axis.line = element_line(color="black", linewidth=0.5),

axis.ticks = element_line(color="black", linewidth=0.5),

axis.text = element_text(color="black"),

plot.title = element_text(face="bold", hjust=0)

)

}

\# -------------------------

\# Combine 3D7 plots

\# -------------------------

figure_3D7 <- (pA_3d7 | pB_3d7) /

(pC_3d7 | pD_3d7)

ggsave("3D7_Combined_Figure.png",

figure_3D7,

width = 7, height = 7, dpi = 600)

\# -------------------------

\# Combine DD2 plots

\# -------------------------

figure_DD2 <- (pA_dd2 | pB_dd2) /

(pC_dd2 | pD_dd2)

ggsave("DD2_Combined_Figure.png",

figure_DD2,

width = 7, height = 7, dpi = 600)

**R SCRIPT — Combine All 8 Plots Into One Figure**

library(ggplot2)

library(patchwork)

\# -------------------------

\# Nature-style theme

\# -------------------------

theme_nature <- function() {

theme_bw(base_size = 10) +

theme(

panel.grid = element_blank(),

panel.border = element_blank(),

axis.line = element_line(color="black", linewidth=0.5),

axis.ticks = element_line(color="black", linewidth=0.5),

axis.text = element_text(color="black"),

plot.title = element_text(face="bold", hjust=0)

)

}

\# -------------------------

\# Combine all 8 plots

\# Layout:

\# 3D7 (A–D) on top row

\# DD2 (E–H) on bottom row

\# -------------------------

figure_all <- (pA_3d7 | pB_3d7 | pC_3d7 | pD_3d7) /

(pA_dd2 | pB_dd2 | pC_dd2 | pD_dd2)

\# -------------------------

\# Save combined figure

\# -------------------------

ggsave("Combined_3D7_DD2_All8.png",

figure_all,

width = 12, height = 6, dpi = 600)
